# Temporary Knockdown of p53 During Focal Limb Irradiation Increases the Development of Sarcomas

**DOI:** 10.1158/2767-9764.CRC-23-0104

**Published:** 2023-12-05

**Authors:** Andrea R. Daniel, Chang Su, Nerissa T. Williams, Zhiguo Li, Jianguo Huang, Omar Lopez, Lixia Luo, Yan Ma, Lorraine da Silva Campos, Sara R. Selitsky, Jennifer L. Modliszewski, Siyao Liu, Rosa Hernansaiz-Ballesteros, Yvonne M. Mowery, Diana M. Cardona, Chang-Lung Lee, David G. Kirsch

**Affiliations:** 1 Department of Radiation Oncology, Duke University Medical Center, Durham, North Carolina.; 2 Department of Biostatistics and Bioinformatics, Duke University Medical Center, Durham, North Carolina.; 3 QuantBio LLC, Durham, North Carolina.; 4 Tempus Labs, Inc., Chicago, Illinois.; 5 Department of Head and Neck Surgery & Communication Sciences, Duke University Medical Center, Durham, North Carolina.; 6 Department of Pathology, Duke University Medical Center, Durham, North Carolina; 7 Department of Pharmacology and Cancer Biology, Duke University Medical Center, Durham, North Carolina.

## Abstract

Approximately half of patients with cancer receive radiotherapy and, as cancer survivorship increases, the low rate of radiation-associated sarcomas is rising. Pharmacologic inhibition of p53 has been proposed as an approach to ameliorate acute injury of normal tissues from genotoxic therapies, but how this might impact the risk of therapy-induced cancer and normal tissue injuries remains unclear. We utilized mice that express a doxycycline (dox)-inducible p53 short hairpin RNA to reduce *Trp53* expression temporarily during irradiation. Mice were placed on a dox diet 10 days prior to receiving 30 or 40 Gy hind limb irradiation in a single fraction and then returned to normal chow. Mice were examined weekly for sarcoma development and scored for radiation-induced normal tissue injuries. Radiation-induced sarcomas were subjected to RNA sequencing. Following single high-dose irradiation, 21% of animals with temporary p53 knockdown during irradiation developed a sarcoma in the radiation field compared with 2% of control animals. Following high-dose irradiation, p53 knockdown preserves muscle stem cells, and increases sarcoma development. Mice with severe acute radiation-induced injuries exhibit an increased risk of developing late persistent wounds, which were associated with sarcomagenesis. RNA sequencing revealed radiation-induced sarcomas upregulate genes related to translation, epithelial–mesenchymal transition (EMT), inflammation, and the cell cycle. Comparison of the transcriptomes of human and mouse sarcomas that arose in irradiated tissues revealed regulation of common gene programs, including elevated EMT pathway gene expression. These results suggest that blocking p53 during radiotherapy could minimize acute toxicity while exacerbating late effects including second cancers.

**Significance:**

Strategies to prevent or mitigate acute radiation toxicities include pharmacologic inhibition of p53 and other cell death pathways. Our data show that temporarily reducing p53 during irradiation increases late effects including sarcomagenesis.

## Introduction

The p53 tumor suppressor protein is a critical component of the cellular DNA damage response machinery that drives cell death in acutely responding irradiated tissues (1). Following DNA damage, p53 initiates cell-cycle arrest, apoptosis, or senescence in a cell type–dependent manner. Because p53-mediated cell death causes hematologic and other acute radiation and chemotherapy toxicities, temporarily blocking p53 during genotoxic therapies for patients with p53-mutant tumors has been proposed as a viable approach to prevent acute side effects of cancer therapy (1, 2). Preventing the death of cells injured by radiation is a strategy that has also been employed for the development of countermeasures to mitigate the acute radiation syndrome following radiological disasters, theorizing that inhibiting programed cell death pathways will prevent the death of heavily damaged cells and preserve tissue integrity (3). While temporary inhibition of p53 or other cell death pathway components may spare sensitive normal tissues from radiation injury, it is also possible that by preventing the death of cells damaged by radiation late effects, including cancer development, may be exacerbated.

Approximately half of all patients with cancer receive radiotherapy as part of their treatment. Radiation exposure, either from radiotherapy in the clinic, a radiation accident, or radiological warfare, is a primary risk factor for the development of sarcoma (4, 5). Cancer survivorship rates are increasing as a result of improved cancer therapy, and as a corollary, rates of treatment-related malignancies are rising (6). Because children and young adults who survive cancer have many years to live after radiotherapy exposure, they are at an increased risk of developing radiation-induced sarcomas relative to older adults. Treatment-related secondary sarcomas are often aggressive and more challenging to treat than *de novo* tumors (4, 6).

Sarcomas are life-threatening tumors that occur in adults, young adults, and comprise approximately 15% of all childhood tumors. Sarcomas are heterogeneous and aggressive malignancies that arise from the muscle, fat, bone, or other connective tissues. One of the most common childhood sarcomas, rhabdomyosarcoma, and one of the most common adult sarcomas, undifferentiated pleomorphic sarcoma (UPS), are tumors that can arise from muscle stem/progenitor cells or satellite cells (7).

Our previous work utilizing genetically engineered mice with *in vivo* short hairpin RNA (shRNA) targeting p53 demonstrated that temporarily reducing p53 expression in mice during fractionated low-dose total-body irradiation (TBI) reduced the risk of thymic lymphoma development (8). In this model, reduced p53-mediated apoptosis of bone marrow cells preserved cell competition in the thymic niche to prevent preexisting lymphoma-initiating cells harboring an oncogenic mutation in the thymus from developing into a lymphoma. Thus, persistence of cells damaged by radiation in the p53 knockdown (p53KD) animals leads to reduced tumorigenesis in the lymphoma model. Using the same genetically engineered mice with *in vivo* shRNA to p53, here we demonstrate that temporarily reducing p53 expression in mice during a single high-dose fraction of radiation promotes sarcomagenesis through a cell autonomous mechanism.

## Materials and Methods

### Radiation-induced Sarcoma Mouse Model

All animal procedures for this study were approved by the Institutional Animal Care and Use Committee at Duke University (Durham, NC). Radiation-induced sarcomas were generated as described previously (9). *CMV-rtTA; TRE-p53.1224* and *Actin-rtTA; TRE-p53.1224* mice were kindly provided by Scott Lowe (10). *CMV-rtTA; TRE-p53.1224* and *Actin-rtTA; TRE-p53.1224* mice express a doxycycline (dox)-inducible shRNA against p53 and their littermates harboring only a single allele (either *rtTA* or *TRE-p53.1224*) were used as controls (8). Mice were on a C3H and C57BL/6J mixed genetic background. Six to 24 weeks old mice were placed on a dox diet for 10 days, and then the left hind limb of the mice was irradiated with a single fraction of 30 or 40 Gy. Hind limb irradiation was performed using the X-RAD 225Cx small animal image-guided irradiator (Precision X-Ray). The irradiation field included the whole left hind limb and was defined using fluoroscopy with 40 kVp, 2.5 mA X-rays using a 2 mm Al filter (Supplementary Fig. S1A). Irradiations were performed using parallel-opposed anterior and posterior fields with an average dose rate of 300 cGy/minute prescribed to midplane with 225 kVp, 13 mA X-rays using a 0.3 mm Cu filter. Following irradiation, animals were immediately returned to normal chow.

After irradiation, mice were examined weekly for sarcomas. Upon detection, tumors were harvested with half stored in RNAlater (Thermo Fisher Scientific) for subsequent RNA isolation and half formalin fixed for histologic analysis. Normal muscle samples were collected from contralateral (unirradiated) hind limbs from mice that did or did not develop sarcomas (Supplementary Table S1).

### Satellite Cell Isolation and Flow Cytometry

Muscle satellite cells were isolated from *Pax7-nGFP* (11)*; Actin-rtTA; TRE-p53.1224* mice or littermate controls with *Pax7-nGFP* and only a single allele (either *Actin-rtTA* or *TRE-p53.1224*). Mice were fed dox diet for 10 days, irradiated with a single fraction of 30 Gy to one hind limb, and returned to normal chow. After 48 hours, mice were sacrificed and the muscles from the irradiated and unirradiated hind limbs were collected. Muscle satellite cells were isolated using a published protocol (12). Two million muscle cells were stained with propidium iodide and subjected to flow cytometric analysis to determine the percentage of live GFP+ muscle satellite cells in the irradiated and unirradiated limbs.

### Immunohistochemistry (IHC)

Formalin-fixed (10% neutral buffered formalin) paraffin-embedded tumor tissues were sectioned (5 µm thick) and stained with hematoxylin and eosin (H&E). IHC with antibodies to p53 (Leica, NCL-L-p53-CM5p, RRID:AB_2895247), p-S139 gamma-H2AX (Abcam, ab11174, RRID:AB_297813), and cleaved caspase 3 (Cell Signaling Technology, 9661S, RRID:AB_2341188) were used to characterize irradiated and unirradiated mouse muscles. IHC with antibodies to S100 (Dako, GA504, RRID:AB_2811056), Myod1 (Dako, M3512, RRID:AB_2148874), Myogenin (Dako, IR06761-2), Desmin (Dako, M0760, RRID:AB_2335684), Cytokeratin (Abcam, ab9377, RRID:AB_307222), CD31(Abcam, ab28364, RRID:AB_726362), SMA (Abcam, ab5694, RRID:AB_2223021), and CD45 (BD Biosciences, 553076, RRID:AB_394606) were used to characterize tumor cell lineage and diagnosis. Sections from radiation-induced injured limbs were subjected to trichrome staining (Abcam, ab150686). Tumor and injury histology slides were reviewed by D.M. Cardona, an expert sarcoma pathologist, while masked to mouse genotype and treatment.

### Injury Scoring

The irradiated limbs of mice were examined weekly and radiation-induced injuries were scored on the basis of a system adapted from the Douglas-Fowler Skin Reaction Scoring system (13) as detailed in Supplementary Table S2. Note, evaluation of limb injuries was added to the study when high levels of tissue damage were observed in cohorts of mice irradiated early in the study. Therefore, complete early injury scoring profiles were not recorded for all mice in the study and these animals were excluded from the relevant analyses.

### Generation of p53/RB-Mutant Tumors

The pX334-dual single-guide RNAs (sgRNA) backbone vector was constructed by deletion of the Cre gene in the pX333-Cre vector (14) using two NcoI sites by standard cloning methods. For cloning pX334-sgTrp53, the pX334-dual sgRNAs vector was digested with BsaI enzyme and ligated to annealed sgRNA oligonucleotides targeting mouse Trp53 (Trp53 sgRNA: GTGTAATAGCTCCTGCATGG) (15). For cloning four individual pX334-sgTrp53-sgRb1 vectors, the pX334-sgTrp53 vector was digested with Bbs1 enzyme and ligated to annealed sgRNA oligonucleotides targeting four different loci of mouse Rb1, respectively (Rb1 sgRNA_1: AAATGATACGAGGATTATCG; Rb1 sgRNA_2: AGAGAAGTTTGCTAACGCTG; Rb1 sgRNA_3: TAAGTACGTTCAGAATCCAC; Rb1 sgRNA_4: GCAGTATGGTTACCCTGGAG; ref. 16). Next, 50 µg of four equally mixed pX334-sgTrp53-sgRb1 plasmids were intramuscularly injected into at least 6-week-old Rosa26^LoxP-Cas9-EGFP/LoxP-Cas9-EGFP^ mice with constitutive expression of Cas9 through *in vivo* electroporation as described previously (14). After injection, mice were examined weekly for sarcomas. Upon detection, tumors were harvested with half submerged in RNAlater (Thermo Fisher Scientific) for subsequent RNA isolation.

### RNA Isolation and Sequencing

Total RNA was isolated from tumors and normal muscle using Direct-zol RNA Miniprep Kit (Zymo Research). RNA samples were sequenced at the Duke Center for Genomic and Computational Biology Shared Resource. Total RNA was sequenced using paired end 150 bp reads. A total of 100 million reads were sequenced per sample in triplicate on a NovSeq instrument.

### Gene Expression Analysis of Radiation-induced Mouse Sarcomas

RNA sequencing (RNA-seq) reads were aligned to mm10 mouse genome reference using STAR v.2.7.6a (17). Transcripts were quantified using Salmon v1.4.0 (18). Docker image used for alignment found on Dockerub: unclineberger/rna-seq-quant:2.3. Counts were normalized using DESeq2 (19). After normalization and transformation, counts were utilized in principal components analysis for the purpose of identifying outliers. Genes were included in downstream analyses if they had at least 10 reads in any one sample. Differential expression analyses were performed with DESeq2. The Benjamini–Hochberg method was utilized to adjust *P* values for multiple comparisons. Gene set enrichment analysis (GSEA) was performed with the mouse Hallmark (“mh”) and curated pathways (“m2”) gene sets obtained from Molecular Signatures Database (MSigDB) utilizing the fgsea package. Immune module gene sets from Charoentong and colleagues (2017) and Bindea and colleagues (2013; refs. 20, 21) were converted to mouse gene symbols by sentence case conversion. The immune infiltration scores were computed by calculating median values for all genes in the set after scaling the normalized counts.

### Gene Expression Analysis of Human and Mouse Radiation-associated and Sporadic UPS

RNA-seq data from the 14 mouse radiation-induced UPS (excluding non-UPS tumors S14 and S19; Supplementary Table S1) were compared with nine p53/RB sarcomas. The mouse radiation-induced tumors were sequenced in the same run as two of the nine p53/RB sarcomas. Subsequently, all nine of the p53/RB tumors were subjected to RNA-seq, including resequencing of the two samples sequenced with the radiation-induced sarcomas. These two samples served to anchor the analysis of the two RNA-seq datasets. We applied five different batch correction methods: limma (22), Combat, Combat-Seq (23), median adjustment, and DWD. All batch correction methods were applied after normalizing the raw counts using DESeq2 vst() function (19). All batch correction methods except for DWD appeared to overcorrect the data, that is, bringing the two biological groups closer to each other instead of the matched samples. In addition, the differential expression analysis of the DWD adjusted data using limma showed that about half of the genome was differentially expressed between p53\RB sarcomas and radiation-induced sarcomas, indicating identification of many false positive genes. The unadjusted data already separated the samples by biological group, keeping the shared samples in the batches nearby. Thus, no batch correction was applied, and the unadjusted data were used for downstream analysis. The two shared samples between batches were collapsed using the mean. DESeq2 was then used to find differentially expressed genes between p53\RB sarcomas and radiation-induced sarcomas. GSEA was performed using R/fgsea and mouse hallmark gene sets obtained from the MSigDB.

Publicly available RNA-seq datasets from human radiation-associated (GSE102055) and sporadic (GSE71119) UPS were used for analysis (24, 25). For both datasets, the processed RNA-seq counts for Gene Expression Omnibus was used. The genes in GSE71119 were filtered for those that had a maximum >−9.965784 and in GSE102055 the genes were filtered with a median >0. To combine the datasets, we gene-scaled and then combined, assessing the level of overlap using principal components analysis. Next, differential gene expression analysis was performed using a generalized linear model. We computed pathway enrichment using fGSEA with linear model estimates as weights. To compare the public human dataset with the mouse dataset, we plotted the normalized enrichment score for each gene set in the mouse model against the human model. Per sample gene set score was computed using R/gsva.

### Data Availability

The RNA-seq data generated in this study are publicly available in the Sequence Read Archive, SUB12211398.

## Results

### Temporary Reduction in p53 Expression During Irradiation Increases Sarcomagenesis

**FIGURE 1:**
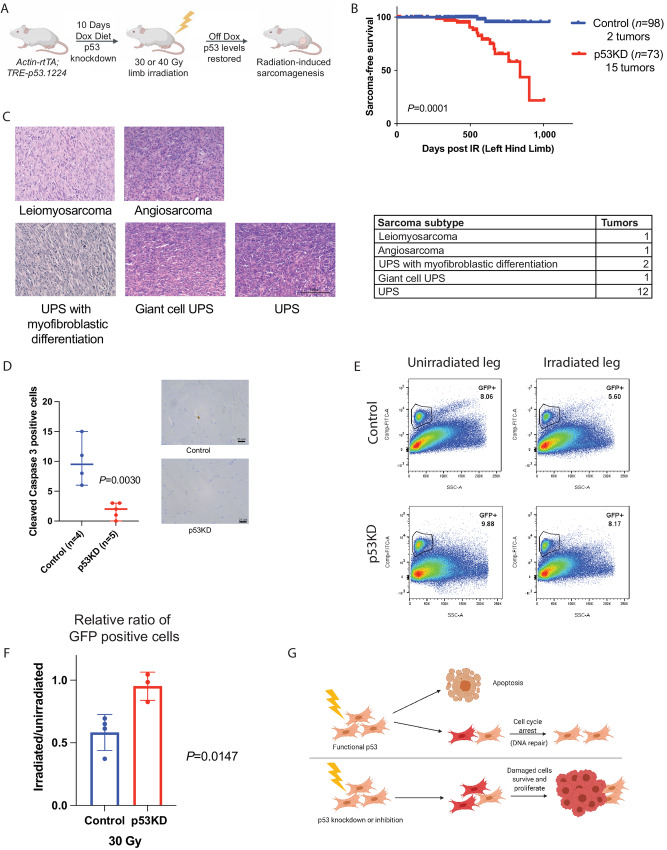
Temporary reduction in p53 expression during irradiation increases sarcomagenesis. **A,** Schematic showing mice fed dox diet for 10 days to drive expression of shRNA to knockdown p53, irradiated with 30 or 40 Gy to the hind limb, returned to normal chow, and followed for sarcoma development and normal tissue injury in the radiation field. **B,** Kaplan–Meier curves show radiation-induced sarcoma-free survival of control and p53KD mice irradiated with 30 or 40 Gy to the hind limb. *P* value is from a log-rank test. **C,** Representative images of a H&E-stained radiation-induced sarcoma subtypes and table with the number of tumors from each subtype. **D,** Quantitation and representative IHC images of mouse muscle tissue from control and p53KD mice 4 hours after 30 Gy irradiation stained with antibodies recognizing cleaved caspase 3. The number of positive cells per tissue section is graphed. **E,** Representative flow cytometry dot plots of GFP+ muscle satellite cells isolated from unirradiated (left) and irradiated (right) limbs from control (top) or p53KD (bottom) mice. **F,** The relative ratio of GFP+ cells in the irradiated limb (30 Gy) over the unirradiated limb is graphed (±SEM). Each dot represents one mouse. *P* value is from a *t* test. **G,** Schematic showing that cells with functional p53 undergo apoptosis or cell-cycle arrest and repair after irradiation, while damaged cells with impaired p53 function are protected from cell death and persist. Figure generated using BioRender.

We and others previously demonstrated that a single fraction of high-dose irradiation (10–70 Gy) to the hind limb of wild-type mice can lead to radiation-induced sarcomas in a small percentage of mice (9, 26). Furthermore, sarcomas can develop following low-dose irradiation (1–4 Gy in single fraction) in mice deficient in p53 (27, 28). Notably, the p53 pathway is inactivated at a high frequency in human radiation-associated tumors (29), which suggests that loss of p53 function may play an important role in initiation and/or maintenance of radiation-associated tumors. To investigate whether p53 function during irradiation plays an important role in initiation of radiation-induced sarcomas, we utilized a dox-inducible p53 shRNA mouse model to determine whether temporary knockdown of p53 during irradiation promotes sarcomagenesis (8, 9, 30). *CMV-rtTA; TRE-p53.1224* and *Actin-rtTA; TRE-p53.1224* mice (p53KD) or their littermate controls expressing either only the *rtTA* or *TRE-p53.1224* allele (control) were fed a dox diet for 10 days to knockdown p53 expression and the hind limb was irradiated with a single fraction of 30 or 40 Gy. The mice were immediately returned to normal chow to restore p53 levels within 7 days (8) and followed for sarcoma formation for the entire natural course of their lives (schematic in Fig. 1A). Thirty and 40 Gy doses were chosen to reproducibly generate sarcomas in mice based on published data (26). Recently, stereotactic body radiotherapy in 30 or 34 Gy doses in a single fraction has been prescribed for patients with non–small cell lung cancer, thus this is a clinically relevant dosing scheme for some patients (31). Many patients receive 50–70 Gy of fractionated radiation in the clinic. Importantly, we published that radiation-induced mouse tumors in this model (9) display similar genetic features to published human radiation-associated tumors that develop following fractionated radiotherapy treatments (32, 33).

To evaluate dox-mediated induction of p53 shRNA, we harvested hind limb muscles from p53KD mice following 10 days on dox diet and detected a significant induction of shp53.1224 by qRT-PCR compared with control mice on dox diet (Supplementary Fig. S1B). To confirm p53KD, similar cohorts of mice were placed on dox diet for 10 days and irradiated with 30 Gy to the hind limb, muscles were harvested after 4 hours and reduced p53 levels were observed by IHC in the p53KD group (39% positive nuclei) compared with the control group (74% positive nuclei; Supplementary Fig. S1C). Of the 40 mice with p53 temporarily knocked down during 30 Gy irradiation, 8 developed sarcomas in the radiation field (20%), while no tumors were detected in the 48 control mice that received 30 Gy irradiation (Supplementary Fig. S2A). Similarly, 7 of the 32 (22%) p53KD animals that received 40 Gy to the hind limb developed in-field sarcomas compared with 2 of 49 (4%) control mice (Supplementary Fig. S2C). In total, 15 of the 73 (20.5%) irradiated p53KD animals and 2 of the 98 (2%) control animals developed radiation-induced sarcomas (Fig. 1B). Notably, despite the increased development of sarcomas in the p53KD group, there was no significant difference in the overall survival between the p53KD and control groups at either radiation dose (Supplementary Fig. S2B and S2D). Interestingly, male p53KD animals irradiated with 30 or 40 Gy developed more radiation-induced sarcomas compared with female p53KD animals (Supplementary Fig. S2E). Additional cohorts of mice that did not receive dox were irradiated with 30 Gy to the hind limb to control for potentially aberrant expression of p53 shRNA independent of dox regulation. No significant difference was observed in sarcoma development within the radiation field between the mice harboring the two-allele system (*rtTA* and *TRE-p53.1224* alleles) for inducible p53KD and the control mice with only one allele (either the *rtTA* or *TRE-p53.1224* allele; Supplementary Fig. S2E). Furthermore, cohorts of mice harboring the two-allele or one-allele system without irradiation were followed for their lifetime and no limb sarcomas were detected (Supplementary Fig. S2F).

The radiation-induced sarcomas developed in mice between 269 and 903 days after irradiation with a median latency of 553 days (Supplementary Table S1). Review of the H&E-stained tumor sections and corresponding IHC analysis revealed that 13 of the 17 sarcomas were classified as UPS, two were designated as UPS with myofibroblastic differentiation, one as a leiomyosarcoma, and one as an angiosarcoma (Fig. 1C).

### Temporary Knockdown of p53 During Irradiation Preserves Muscle Satellite Cells

In our prior work, we performed whole-exome sequencing (WES) on a cohort of radiation-induced sarcomas from this mouse model and determined that while these sarcomas exhibited a relatively low nonsynonymous somatic mutational burden compared with carcinogen-induced sarcomas, they exhibited a distinct genetic signature characterized by C-to-T mutations that is indicative of oxidative damage (9). The radiation-induced mouse sarcomas also displayed a high indel to substitution ratio and a frequent gene copy-number variations (CNV; ref. 9) which is consistent with the types of genetic damage observed in human radiation-associated sarcomas (24, 32). These data led us to hypothesize that the increased rate of sarcomagenesis in the irradiated p53KD animals, compared to controls with wild-type p53 levels, may be due to protection from radiation-induced p53-mediated cell death of tumor-initiating cells. Cells expressing wild-type levels of p53 that exhibit extensive radiation damage may undergo p53-mediated apoptosis (34). To evaluate DNA damage in irradiated muscles of control and p53KD mice, we placed mice on dox for 10 days, irradiated the hind limb with 30 Gy and harvested muscles for IHC 4 hours later. We observed a substantial induction in phospho-gamma-H2AX staining in irradiated control and p53KD muscles indicating high levels of DNA damage (Supplementary Fig. S1D). Cleaved caspase 3 positive cells were also evaluated as a marker of p53-mediated apoptosis signaling. Irradiated muscles from control mice exhibited significantly elevated numbers of cleaved caspase 3 positive cells compared with irradiated muscles from p53KD animals (Fig. 1D). When expression of p53 is temporarily reduced or inhibited during irradiation, the damaged cells may survive and initiate sarcomas. Because many of the radiation-induced tumors in our model are UPS tumors and because we and others have previously used Pax7-CreER mice to show that mouse sarcomas that mimic human UPS can arise from Pax7+ myogenic progenitor cells (7, 35), we examined the fate of satellite cells in mice with temporary p53 KD during irradiation. Satellite cells express the muscle lineage marker Pax7. To label muscle satellite cells in our model, we crossed the p53KD mice to Pax7-nGFP mice (11). Mice expressing nGFP in Pax7+ cells were placed on dox diet for 10 days to knockdown p53, irradiated with 30 Gy to one hind limb, and then returned to normal chow. After 48 hours, when satellite cells are ablated after high-dose irradiation (36), muscles from the irradiated and the unirradiated hind limbs of each mouse were collected and muscle stem cells were isolated. Flow cytometry for GFP+ cells was performed to determine the relative percentage of live satellite cells remaining in the muscle 48 hours after irradiation (Fig. 1E). The relative ratio of GFP+ cells remaining in the irradiated leg compared with the unirradiated leg of each mouse was determined (Fig. 1F). In the p53KD mice, there was minimal loss of GFP+ muscle satellite cells after irradiation, while approximately 40% of GFP+ muscle satellite cells were lost in the control mice. A similar experiment was performed using 18 Gy to one hind limb. Again, knockdown of p53 protected against the loss of GFP+ muscle satellite cells after irradiation; however, we observed a variable loss of GFP+ cells in the control group at the lower dose of radiation (Supplementary Fig. S1E). The survival of muscle satellite cells in the p53KD animals after high-dose irradiation supports a model whereby the persistence of damaged irradiated cells, that would have undergone p53-mediated apoptosis, may initiate oncogenic transformation and eventual sarcoma formation (Fig. 1G, schematic).

### Temporary Reduction in p53 Expression During Irradiation Promotes Chronic Injuries

The strategy of inhibiting p53 during genotoxic therapies to reduce acute side effects may exacerbate other late effects of radiation in addition to carcinogenesis. The consequence of p53-mediated signaling in response to radiation is cell- and tissue-type dependent. For example, loss of p53 may block apoptosis and/or promote mitotic death (8, 37). Therefore, the probability of developing late effects of hind-limb irradiation following p53 knockdown may vary with tissue type (i.e., muscle, skin, vasculature, nerves, fat, and bone). Radiation-induced chronic wounds may occur due to an acute wound that fails to heal or may arise months to years after radiation exposure in tissue that initially appears to have recovered from or avoided acute toxicity (38). Late persistent wounds are characterized by inflammation, ulceration, fibrosis, and/or necrosis of soft tissue and bone. Damage to the vasculature of irradiated tissues may contribute to impaired wound healing due to a lack of neovascularization leading to insufficient perfusion (39). We previously showed that p53 is required in endothelial cells to prevent radiation-induced injury to the heart (40).

We observed acute and chronic injuries to the irradiated leg in the control and p53KD mice. Mice were evaluated weekly based on a previously published rubric for skin injury that we adapted to comprehensively assess radiation-induced normal tissue toxicity of the skin, bone, and muscle (ref. 13; Supplementary Table S2). Mice exhibiting signs of injury (skin breakdown and/or swelling) were given a score of 1 and scoring increased with the severity of the injury to a maximum score of 4 (loss of the foot). Acute injuries were defined as occurring within the first 3 months of irradiation, and late injuries were defined as arising after 3 months. Injuries were treated with topical antibiotics and supportive care, including wet food and hydration gel packs provided to injured mice. Late injuries presented as chronic wounds that slowly progressed. Therefore, with few minor exceptions, the last injury score was the highest score the mouse received.

Chronic tissue injuries were examined histologically by a sarcoma pathologist, masked to genotype and treatment, and the degree of fibrosis was assessed using H&E- and trichome-stained slides (Supplementary Fig. S3A–S3F). Tissue from animals scoring a 1–1.75 showed evidence of muscle and skin fibrosis, while those from animals with injury scores between 2 and 2.75 displayed muscle, skin, and neurovascular fibrosis. Half of the tissue injuries scoring 3–3.75 exhibited histologic evidence of skin and muscle fibrosis with an increase incidence of neurovascular fibrosis and some bone remodeling. Animals with an injury score of 4 exhibited histologic evidence of muscle, skin, and neurovascular fibrosis, and bone remodeling (Supplementary Fig. S3G and S3H). Evaluation of injuries in mice receiving 3+ scores from the p53KD and control groups reveal the groups to be similar with slightly more neurovascular fibrosis and slightly less skin fibrosis in the p53KD group (Supplementary Fig. S3I).

**FIGURE 2:**
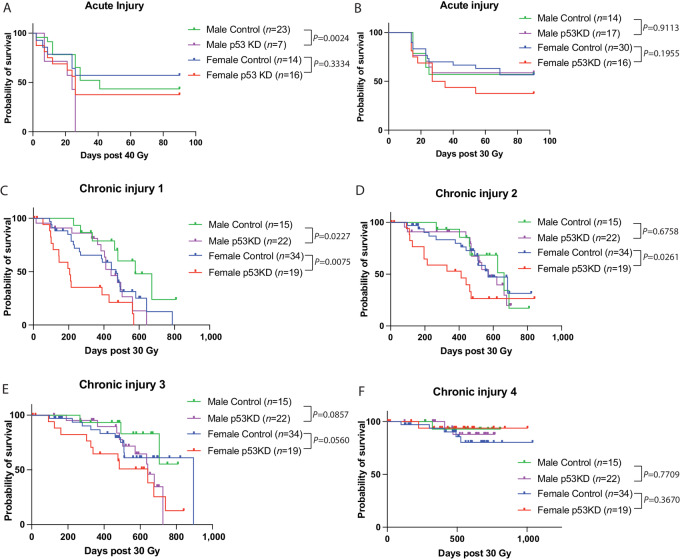
Temporary reduction of p53 during irradiation increases chronic injuries in subsets of mice. **A,** Kaplan–Meier curves show acute injury-free survival (score 1+) of control and p53KD male and female mice irradiated with 40 Gy to the hind limb. *P* value is from a log-rank test. **B,** Kaplan–Meier curves show acute injury-free survival (score 1+) of control and p53KD male and female mice irradiated with 30 Gy to the hind limb. *P* value is from a log-rank test. Kaplan–Meier curves show chronic injury-free survival from scores 1+ (**C**), 2+ (**D**), 3+ (**E**), or 4 (**F**) of control and p53KD male and female mice irradiated with 30 Gy to the hind limb. *P* value is from a log-rank test.

Prior work has shown meaningful yet small differences in radiation sensitivities by sex, therefore we evaluated radiation injuries in male and female mice in our study (41). Examination of acute injuries scoring at least a 1 demonstrated a higher frequency of injuries in the p53KD group compared with control in the male animals that received 40 Gy hind limb irradiation only (Fig. 2A), but no significant difference was observed in the animals that received 30 Gy (Fig. 2B). In contrast, the development of chronic injuries was significantly greater in the female p53KD animals that received 30 Gy compared with female control animals for score levels 1 and 2, but not 3 and 4 (Fig. 2C–F). Male p53KD mice developed significantly more level 1, but not level 2–4, injures compared with male control mice. Female p53KD mice also develop significantly more level 1 injuries than male control and p53KD mice. For animals that received 40 Gy, no significant differences in chronic injuries were observed between males and females at any score level. A significant increase in chronic injuries in the p53KD group compared with control was only observed for the score level 2 (Supplementary Fig. S4A–S4D). The combined (30 and 40 Gy) control groups exhibited a median final injury score of 1.375 compared with 2.375 from the p53KD animals, and this difference did not reach statistical significance (Supplementary Fig. S4E). Notably, in the control cohorts that did not receive dox prior to 30 Gy irradiation, no significant difference was observed between the genotypes at any score level (Supplementary Fig. S5).

We next performed a correlation coefficient test comparing the acute and chronic injury scores from all the mice, which included the p53KD and control mice that received 30 or 40 Gy (Supplementary Fig. S4F). This analysis showed an association between mice with a high acute injury score and the development of a high chronic injury score. However, low acute injury score was not associated with low or high chronic injury score.

### Radiation-induced Chronic Injuries Increase The Risk of Sarcomagenesis

**FIGURE 3:**
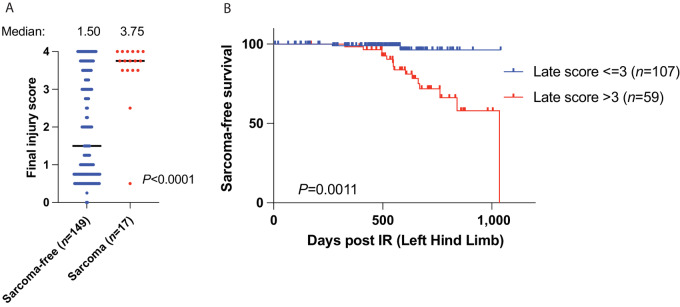
Radiation-induced chronic injuries increase the risk of sarcomagenesis. **A,** The final injury scores of the control and p53KD mice that received 30 or 40 Gy are plotted. The final injury scores of mice that did not develop a radiation-induced sarcoma (blue) are compared with the scores of mice that did develop a radiation-induced sarcoma (red). *P* value is from a *t* test. **B,** Kaplan–Meier curves show radiation-induced sarcoma-free survival of the control and p53KD mice irradiated with 30 or 40 Gy to the hind limb. Mice with chronic injury scores equal to or less than 3 are compared with mice with chronic injury scores greater than 3. *P* value is from a cox proportional hazards model.

Understanding the relationship between tissue damage and tumor promotion may improve secondary cancer detection and prevention strategies (42). We previously observed that muscle tissue injury promotes sarcoma development by stimulating muscle satellite cell activation (43). In addition, chronic inflammation and wound healing can create a tumor promoting microenvironment with a permissive immune milieu (44, 45) and epigenetic reprogramming to stimulate tumor outgrowth (46). We compared the final chronic injury scores of mice from the p53KD and control groups receiving 30 or 40 Gy that developed a radiation-induced sarcoma to those of mice that did not develop a sarcoma in the radiation field (Fig. 3A). The sarcoma-free mice had a median chronic injury score of 1.5, while the median score of the sarcoma-bearing mice was significantly higher at 3.75. Of the 17 animals that developed radiation-induced sarcomas, 15 exhibited injury scores greater than 3. Animals in this group (*n* = 15) were observed to have a level 3+ injury for 29 to 455 days (median 132 days) prior to tumor detection and all but 1 sarcoma-bearing mice spent several days with limb injuries (Supplementary Fig. S6A). Notably, mice that developed radiation-induced sarcomas reached the injury score of 2 and 3 earlier than mice that did not go on to develop radiation-induced sarcomas (Supplementary Fig. S6B and S6C). A Cox model analysis demonstrated that, independent of the covariants dose and genotype, radiation-induced chronic injuries (score 3+) are associated with sarcomagenesis (Fig. 3B; Supplementary Fig. S6D).

### Radiation-induced Sarcomas Exhibit an Increase in Proliferative Gene Expression Programs and a Decrease in Myogenic Differentiation Gene Expression Programs

**FIGURE 4:**
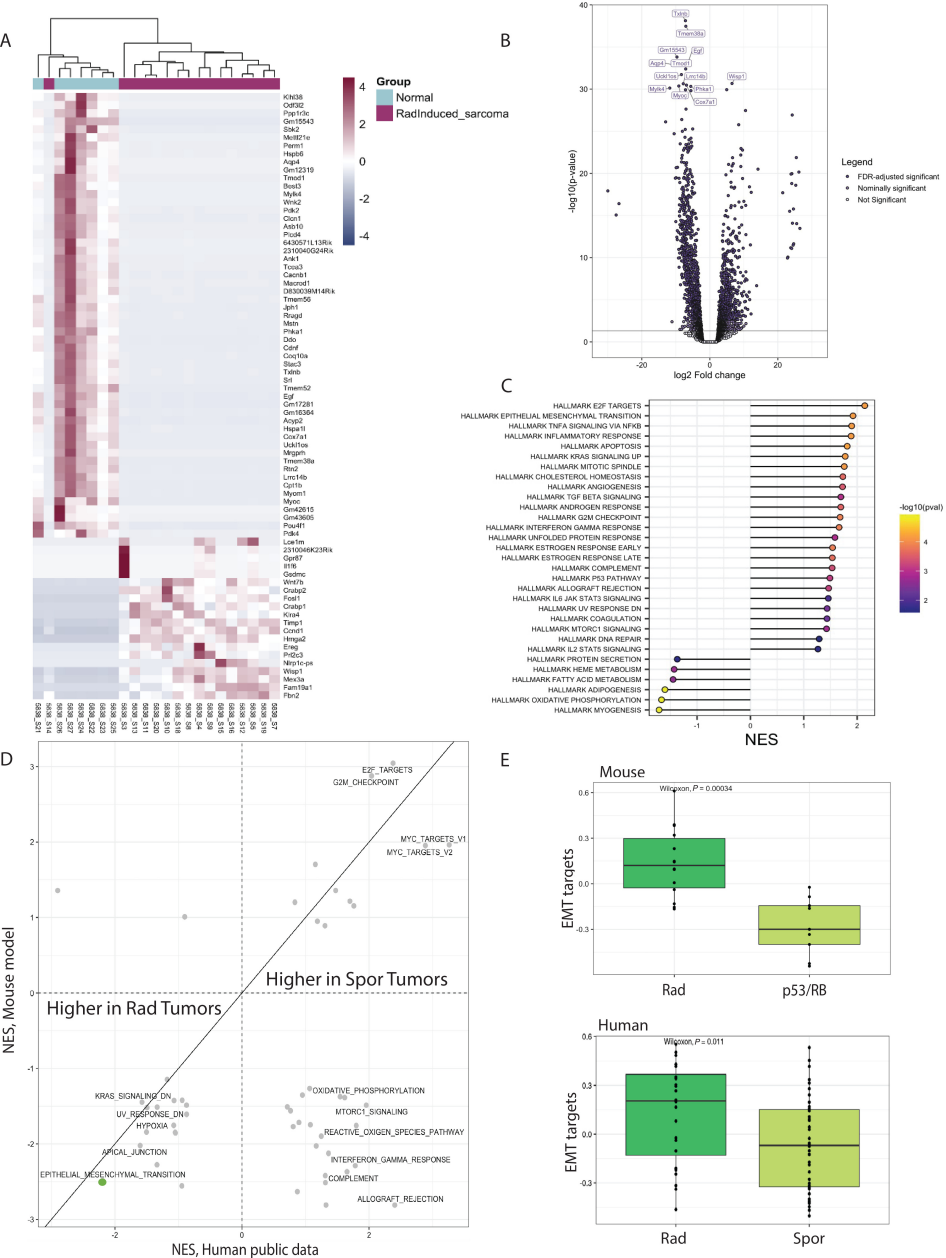
Radiation-induced sarcomas exhibit an increase in proliferative gene programs and a decrease in myogenic differentiation programs. **A,** Heat map of the top 75 differentially expressed genes in radiation-induced sarcomas (*n* = 16) versus normal muscle (*n* = 7). Genes (rows) are colored by scaled, normalized expression values. Both rows and columns are clustered. **B,** Volcano plot of log_2_ fold change for all genes with base mean greater than 50 in tumor (*n* = 16) versus normal (*n* = 7). Labeled genes have a nominal −log_10_*P* value greater than or equal to 30. **C,** Normalized enrichment scores for all FDR significant pathways for GSEA of Hallmark pathways. Points are colored by −log_10_ (nominal *P* value). **D,** Scatter plot comparing the differentially expressed pathways between tumors that arose in irradiated tissues (Rad) versus sporadic (Spor) tumors in mouse (*y*-axis) and human (*x*-axis) datasets. The EMT pathway is highlighted with a green dot. **E,** Boxplot showing gene expression of EMT pathway targets in mouse radiation-induced UPS compared with p53/RB tumors (top) and in human radiation-associated undifferentiated sarcomas compared with sporadic undifferentiated sarcomas.

We do not currently have robust methods to determine whether second malignancies in patients were initiated because of prior radiotherapy. Identifying a gene expression signature of radiation-associated cancer in human samples is difficult due to variation in the radiotherapy prescription and the irradiated tissue volume and type. Conversely, our well-controlled mouse model of radiation-induced sarcoma provides a system to examine expression and pathway signatures driving radiation-induced tumorigenesis. In our prior analysis of WES data from radiation-induced sarcomas, no recurrent nonsynonymous somatic mutations in oncogenes or tumor suppressor genes were identified (9). To gain insight into the mechanisms of radiation sarcomagenesis, we analyzed RNA-seq results from 16 radiation-induced sarcomas in our cohort and normal muscles from 7 age-matched littermate mice (117–840 days old, median age 343 days; Supplementary Table S1). As an additional control, we also performed RNA-seq on two sarcomas initiated by loss of the *p53* and *Rb1* tumor suppressor genes. Principal component analysis revealed that the sarcoma samples clustered together, and the normal muscle samples clustered as a group (Supplementary Fig. S7A). Upon examination of the top 75 differentially expressed genes, the 14 UPS, and one leiomyosarcoma clustered together (Fig. 4A). Not surprisingly, based on transcriptomic analysis of human radiation-associated sarcomas (24), the angiosarcoma sample did not cluster with the other radiation-induced sarcomas. The radiation-induced sarcomas were characterized by a preponderance of gene downregulation events (*n* = 1,051, *Q* < 0.05) compared with upregulation of genes (*n* = 657, *Q* < 0.05; Fig. 4B). The top downregulated gene was the muscle differentiation marker *MyoC* (ref. 47; Supplementary Fig. S7B), and the top upregulated gene in the radiation-induced sarcomas was the cytokine *Wisp1 (Ccn4)* (Supplementary Fig. S7C), which can promote proliferation (48). Notably, the inflammatory cytokine *Il1f6 (Il36a)* was among the most upregulated genes in the tumors compared with normal muscle (Supplementary Fig. S7D). GSEA revealed that the genes upregulated in tumors were related to epithelial-to-mesenchymal (EMT) transition, inflammation, and cell cycle, while the downregulated pathways were related to myogenesis and metabolism (Fig. 4C; Supplementary Fig. S7E and S7F). The p53/RB sarcomas engineered to lack RB function serve as controls for activated Hallmark E2F target gene expression (Supplementary Fig. S7E). Analysis of immune infiltration in tumors indicated infiltration of natural killer–related cells and evidence of an immune response at the gene level, including significant enrichment of inflammatory cells including mast cells, macrophages, and some T lymphocytes (Supplementary Fig. S8). The tumors also exhibited enrichment of myeloid-derived suppressor cells and regulatory T cells potentially indicating an immunosuppressive microenvironment (21).

Others have examined the transcriptomes of human radiation-associated tumors but found no specific gene expression clustering associated with radiation exposure (24, 49). To evaluate commonalities among murine radiation-induced sarcoma and human radiation-associated sarcoma transcriptional programs we compared our RNA-seq data with published human datasets (24, 25). We first performed differential gene expression analysis on our mouse radiation-induced UPS tumors (*n* = 14) compared with the p53/RB model (*n* = 9) of sporadic sarcomas (Supplementary Fig. S9A–S9F and S9H). A similar analysis was performed comparing the human transcriptome datasets from radiation-associated undifferentiated sarcomas (*n* = 24) and sporadic undifferentiated sarcomas (*n* = 42; Supplementary Fig. S9G and S9I). GSEA revealed concordant differential expression of multiple gene programs, including upregulation of the EMT pathway, in mouse and human sarcomas that developed in irradiated tissue with common histologies (Fig. 4D and E). High EMT pathway expression is consistent with the aggressive nature of radiation-associated sarcomas which exhibit worse treatment outcomes compared with sporadic sarcomas (50).

**FIGURE 5:**
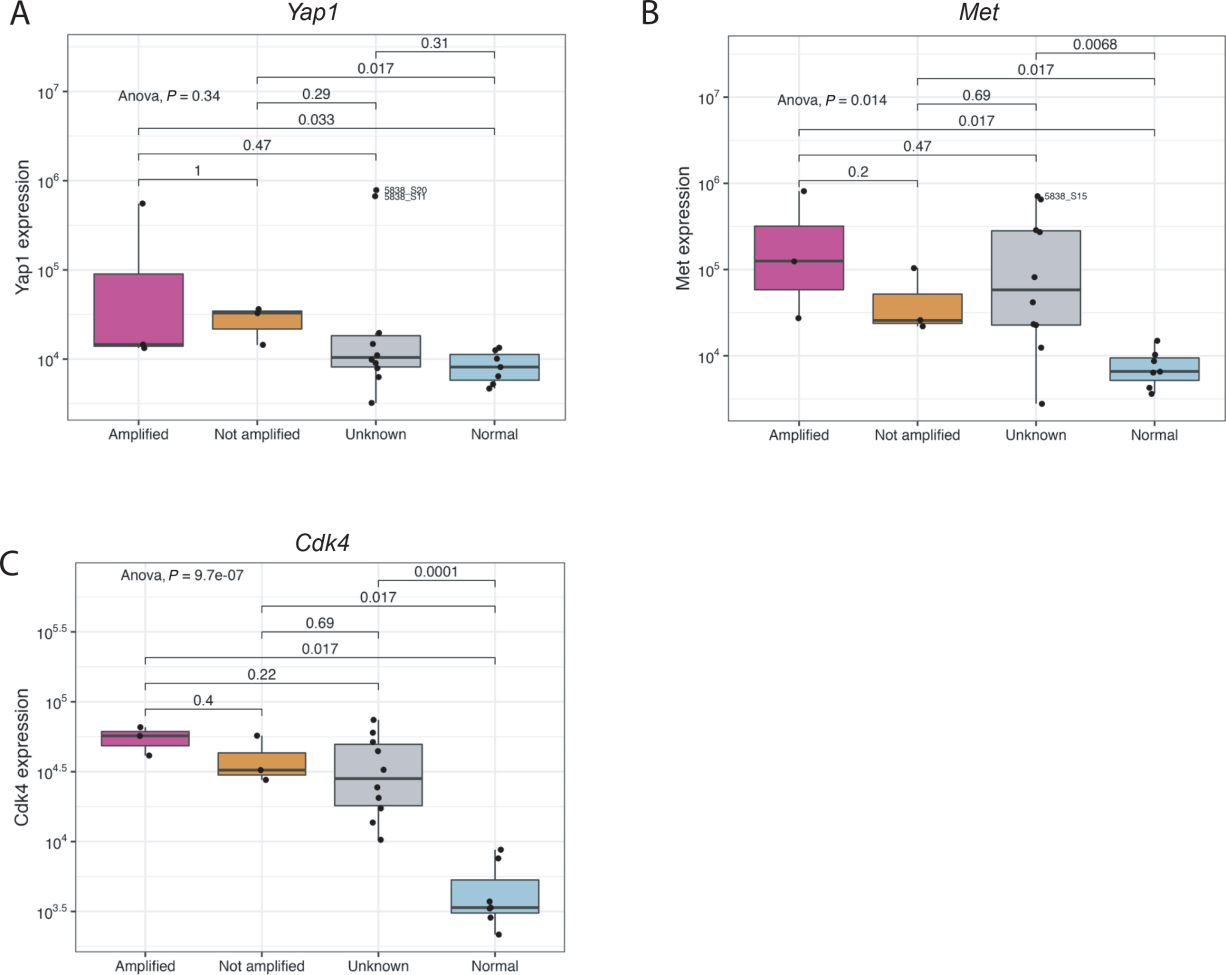
Comparison of CNV and expression of specific oncogenes. Boxplot showing gene expression of *Yap1* (**A**), *Met* (**B**), and *Cdk4* (**C**) in radiation-induced sarcomas where the gene amplification status is known to be amplified (pink), not amplified (orange), or unknown (gray) compared with normal muscle (blue).

Our previous WES data from a subset of the radiation-induced mouse sarcomas (*n* = 7) revealed high copy- CNVs (9) consistent with radiation-induced genetic damage in human tumors (32, 49, 51). We compared the specific oncogene amplification events identified by WES in each sarcoma with the gene expression data from the same tumor (Fig. 5A–C; Supplementary Fig. S10A). *Yap1*, *Met*, and *Cdk4* gene amplification resulted in significant transcriptional overexpression compared with normal muscle. In tumors where *Met* and *Cdk4* were not amplified (*n* = 3) or the amplification status was unknown due to lack of WES (*n* = 10), the expression of these genes was significantly upregulated, suggesting that activation of these oncogenes is selected for during tumor development either by amplification or alternative mechanisms (Fig. 5B and C).

## Discussion

The strategy of blocking p53 to reduce acute radiation-induced tissue injuries and adverse effects from other genotoxic therapies promotes survival of damaged cells to preserve critical cellular functions in radiation-sensitive tissues (2). Therefore, inhibiting p53 during radiotherapy or radiation disaster scenarios has been proposed as an approach to prevent radiation injury. While blocking p53 during radiation exposure can protect the bone marrow from injury (8), our results in mice show that by temporarily blocking p53 during a single high-dose radiation exposure to a limb, late radiation effects, such as chronic tissue injury and radiation-induced sarcomagenesis can be exacerbated. Temporary knockdown of p53 during radiation exposure increased the development of sarcomas harboring genetic hallmarks of radiation damage. One possible explanation for these findings is that cells with severe DNA damage that would have died by p53-mediated cell death instead survived radiation injury due to p53 knockdown to become a sarcoma. As most of the mouse radiation-induced sarcomas mimicked UPS, which can arise from muscle satellite cells (7, 35), we used mice with a GFP reporter allele expressed from the endogenous Pax7 promoter to assess the impact p53 knockdown on the survival of muscle satellite cells after irradiation. We found that a temporary knockdown during a single fraction of 30 Gy to the limb preserved GFP+ cells in the limb, consistent with a model where inhibiting p53 preserves muscle satellite cells with DNA damage that have the potential to become a sarcoma. However, it is possible that temporarily blocking p53 during irradiation may increase radiation-induced sarcomas by other mechanisms.

Tissue sensitivity for radiation-induced tumorigenesis varies by cell type, radiation dose, fractionation (number of radiation exposures), volume of tissue irradiated, and other host factors such as sex, age, and germline mutations. The molecular mechanisms that govern the cellular responses to DNA damage, cell death, and cellular dynamics of tissue regeneration and remodeling impact tissue susceptibility to tumorigenesis (8, 52). The temporary and reversible p53 knockdown system provides a unique model to examine how blocking p53-mediated cell death from irradiation regulates radiation-induced cancer while avoiding permanent p53 loss, which is a well-known driver of radiation-induced cancer (28, 29, 53). Using the same p53KD mouse model, we previously found that radiation-induced lymphoma development in mice following fractionated low-dose TBI is reduced when p53 expression is temporarily abrogated during irradiation (8). Temporary loss of p53 prevented cell death and improved bone marrow cell survival, thus ameliorating acute hematologic toxicity and, surprisingly, lymphoma development, thereby improving overall survival of mice. In the TBI mouse lymphoma model, we demonstrated that temporary p53 knockdown reduces radiation-induced lymphomagenesis by limiting bone marrow cell death thus increasing cell competition for the thymic niche, which prevents the outgrowth of tumor-initiating cells in a non-cell autonomous mechanism. Niche competition is also the mechanism by which bone marrow transplantation prevents radiation-induced thymic lymphoma after TBI (54). Conversely, in this study, we showed that temporarily blocking p53 during high-dose radiation promoted radiation-induced solid tumors, such as sarcomas, in a cell autonomous mechanism. Thus, in the thymic lymphoma model, p53 knockdown-mediated cell survival prevented tumors because surviving cells competed with tumor-initiating cells whereas in the sarcoma model p53 knockdown promoted tumors likely by tumor initiation from damaged surviving cells. Although our results are limited to an experimental system in which blocking radiation-induced cell death occurred by reducing p53, it is conceivable that these findings extend to other approaches to prevent or mitigate cell death from radiation injury. In this scenario, any strategy that promotes the survival of irradiated cells that were destined to die could increase the pool of potential tumor-initiating cells for a radiation-induced cancer. In the setting of a life-threatening radiation disaster, mitigating acute injury to increase the chance of survival would be worth the increased risk of a radiation-induced cancer years later. However, in the context of radiotherapy to treat a cancer, patients may not want an intervention that limits acute radiation toxicity while increasing the risk of radiation-induced tumorigenesis. Regardless, our results underscore the importance of preclinical testing of radiation protectors and mitigators in experiments with high-dose radiation similar to our study so that patients and their physicians will have information on the risk of a radiation modulator potentially exacerbating the risk of developing a radiation malignancy.

In patients, late adverse effects of radiotherapy are frequently irreversible and injuries to irradiated tissue can undergo protracted remodeling and healing phases (55). Irradiated muscle exhibits impaired regenerative capacity despite satellite cell activation and vascular endothelial injury contributes to the development of fibrosis (56). In our study, chronic wounds after high-dose irradiation were characterized by inflammation, tissue fibrosis, tissue atrophy, occluded vasculature, and in severe cases, loss of the limb. Following 30 Gy irradiation, we observed a significant increase in the development of chronic wounds (scores 1–3) in the p53KD group compared with controls. We previously demonstrated that endothelial cells deficient in p53 are sensitized to late effects of radiation (40, 57, 58) which may contribute to reduced healing and increased wound severity. Notably, significant increases in chronic wounds in the p53KD group were only observed in the 40 Gy animals with a score of 2, suggesting that the higher dose of radiation was sufficient to overcome most of the protection afforded to tissues expressing normal levels of p53 following 30 Gy. Sex differences in radiation responses have been observed in human and mouse studies (41), our data revealed sex differences in normal tissue toxicity from radiation and in radiation sarcomagenesis. Further examination of the mechanisms driving sex differences in radiosensitivity is warranted.

Our results reveal commonalties in gene expression pathways among human and murine sarcomas that develop in irradiated tissue. DNA sequencing of the radiation-associated human cancers reveals distinct radiation damage signatures that are also observed in the murine radiation-induced sarcomas (9, 28, 32, 33, 49). In addition to radiation-induced DNA damage through a cell autonomous process, our data suggest that high-dose irradiation may also promote tumor development as a consequence of prolonged tissue injury. Unresolved inflammation creates a permissive microenvironment for malignant conversion, where tumor cells are bathed in progrowth, proremodeling, and proangiogenic signals (44). Furthermore, tissue injury has been shown to accelerate tumor formation via promotion of epigenetic remodeling to induce a chromatin state that regulates gene expression programs favoring neoplastic commitment (46). Our prior studies demonstrated that muscle injury promotes sarcomagenesis though activation of the HGF-Met signaling axis to stimulate satellite cells (43, 59). Interestingly, our RNA-seq data show that *Met* is upregulated in the majority of the radiation-induced sarcomas compared with normal muscle (*n* = 14). Furthermore, radiation-induced sarcomas exhibited an enrichment of inflammatory signaling pathway genes which may result from developing within chronically inflamed tissue.

In sum, the current study demonstrates that temporarily inhibiting p53 during high-dose irradiation of the limb promotes late effects of radiation, including sarcomagenesis. Our findings support a model for radiation sarcomagenesis that results from a combination of radiation-induced DNA damage and a permissive microenvironment from normal tissue injury that promote tumor development. In addition, these results suggest that blocking p53 during radiotherapy for a cancer might increase the risk of developing a radiation-associated malignancy.

## Supplementary Material

Figure S1 Supplementary figure S1 shows irradiated hind limbs of control and p53KD mice

Figure S2 Supplementary figure S2 shows that temporary reduction in p53 expression during irradiation increases sarcomagenesis

Figure S3 Supplementary figure S3 shows histological examination of mouse radiation-induced injuries

Figure S4 Supplementary figure S4 shows high dose irradiation induces chronic injuries in mouse hind limbs

Figure S5 Supplementary figure S5 shows mice without dox treatment sustained radiation-induced injuries in the hind limb

Figure S6 Supplementary figure S6 shows that radiation-induced chronic injuries are associated with sarcomagenesis

Figure S7 Supplementary figure S7 shows gene expression analysis of radiation-induced sarcomas compared to normal muscles

Figure S8 Supplementary figure S8 shows immune infiltration analysis of radiation-induced sarcomas

Figure S9 Supplementary figure S9 shows gene expression analysis of mouse and human sarcomas that arose in irradiated tissue compared to sporadic sarcomas

Figure S10 Supplementary figure S10 show correspondence between gene expression and CNV in radiation-induced sarcomas

Table S1 Supplementary table S1 is the experimental mice table

Table S2 Supplementary table 2 is the injury scoring rubric
