# Supplementary material for: Temporary Knockdown of p53 During Focal Limb Irradiation Increases the Development of Sarcomas
Source: Cancer Res Commun. 2023 Dec 5;3(12):2455–67. doi: 10.1158/2767-9764.CRC-23-0104 (PMC10697056; doi:10.1158/2767-9764.CRC-23-0104)
Supplement: Figure S1 — Supplementary figure S1 shows irradiated hind limbs of control and p53KD mice [file crc-23-0104-s01.pdf]

Figure S1

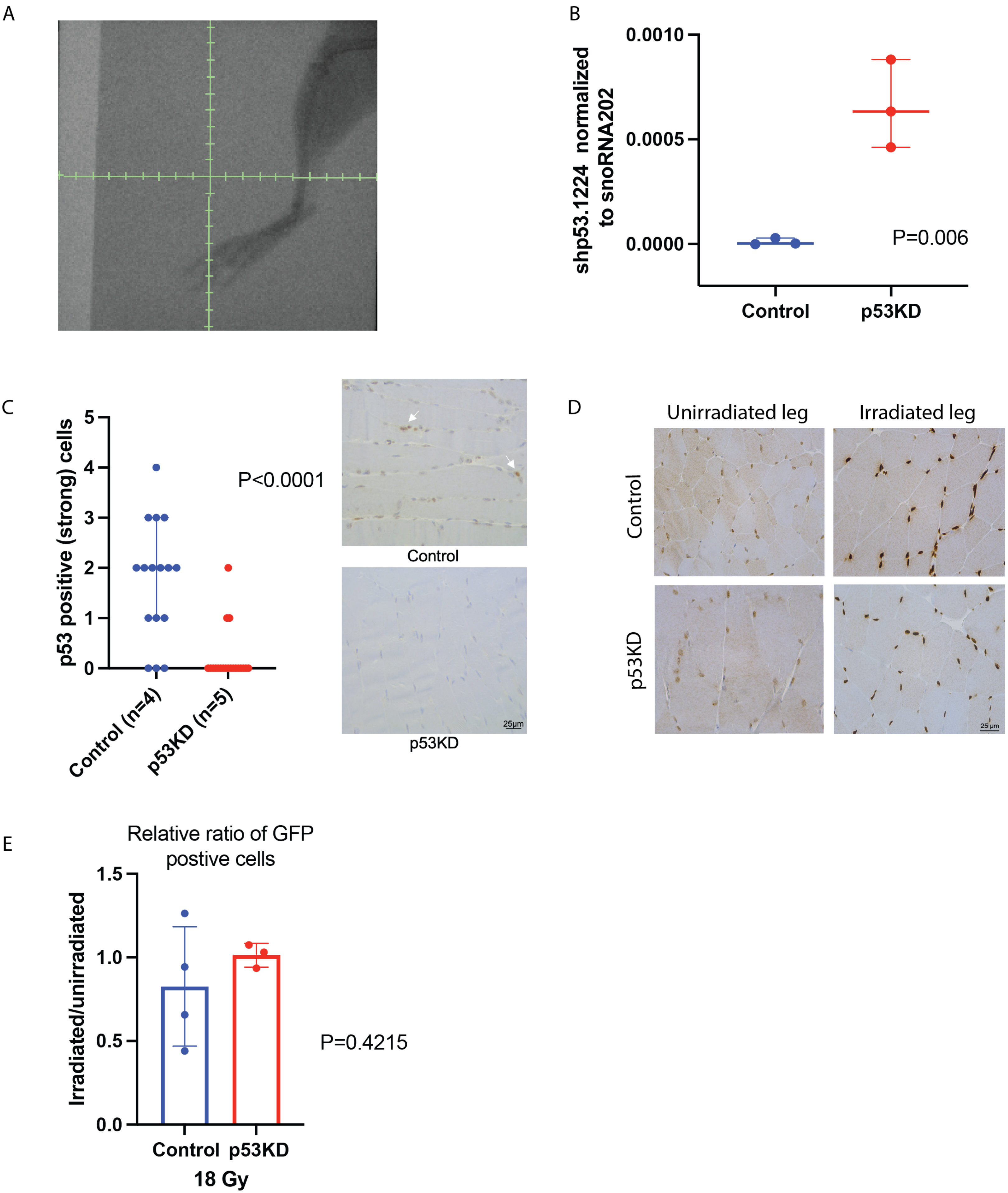

A
B
0.0010
0.0005
0.0000
shp53.1224 normalized to snoRNA202
P=0.006
Control
p53KD
C
p53 positive (strong) cells
5
4
3
2
1
0
P<0.0001
Control (n=4)
p53KD (n=5)
Control
p53KD
25µm
D
Unirradiated leg
Irradiated leg
Control
p53KD
25 µm
E
Relative ratio of GFP postive cells
1.5
1.0
0.5
0.0
Irradiated/unirradiated
P=0.4215
Control
p53KD
18 Gy

Figure S1. Irradiated hind limbs of control and p53KD mice. (A) Irradiations were performed using a small-animal image-guided irradiator and the target was defined using fluoroscopy with 40-kVp, 2.5-mA x-rays using a 2-mm aluminum filter. Representative image of the irradiation field is shown, which includes the whole hind limb. (B) qRT-PCR to evaluate expression of the dox inducible shp53 in hind limb muscle tissue from control and p53KD mice following 10 days on dox diet (n=3). P-value is from a T-Test. (C) Representative IHC images of mouse muscle tissue from control and p53KD mice 4 hours after 30 Gy irradiation stained with antibodies recognizing p53. Quantitation of nuclei with strong positive staining for p53 (white arrows). P-value is from a T-Test. (D) Representative IHC images of mouse muscle tissue from unirradiated and irradiated (4 hours after 30 Gy) control and p53KD mice stained with antibodies recognizing phospho-gamma H2AX. (E) The relative ratio of GFP+ cells in the irradiated limb (18 Gy) over the unirradiated limb is graphed (±SEM). Each dot represents one mouse. P-value is from a T-Test.
