# Supplementary material for: Temporary Knockdown of p53 During Focal Limb Irradiation Increases the Development of Sarcomas
Source: Cancer Res Commun. 2023 Dec 5;3(12):2455–67. doi: 10.1158/2767-9764.CRC-23-0104 (PMC10697056; doi:10.1158/2767-9764.CRC-23-0104)
Supplement: Figure S2 — Supplementary figure S2 shows that temporary reduction in p53 expression during irradiation increases sarcomagenesis [file crc-23-0104-s02.pdf]

Figure S2

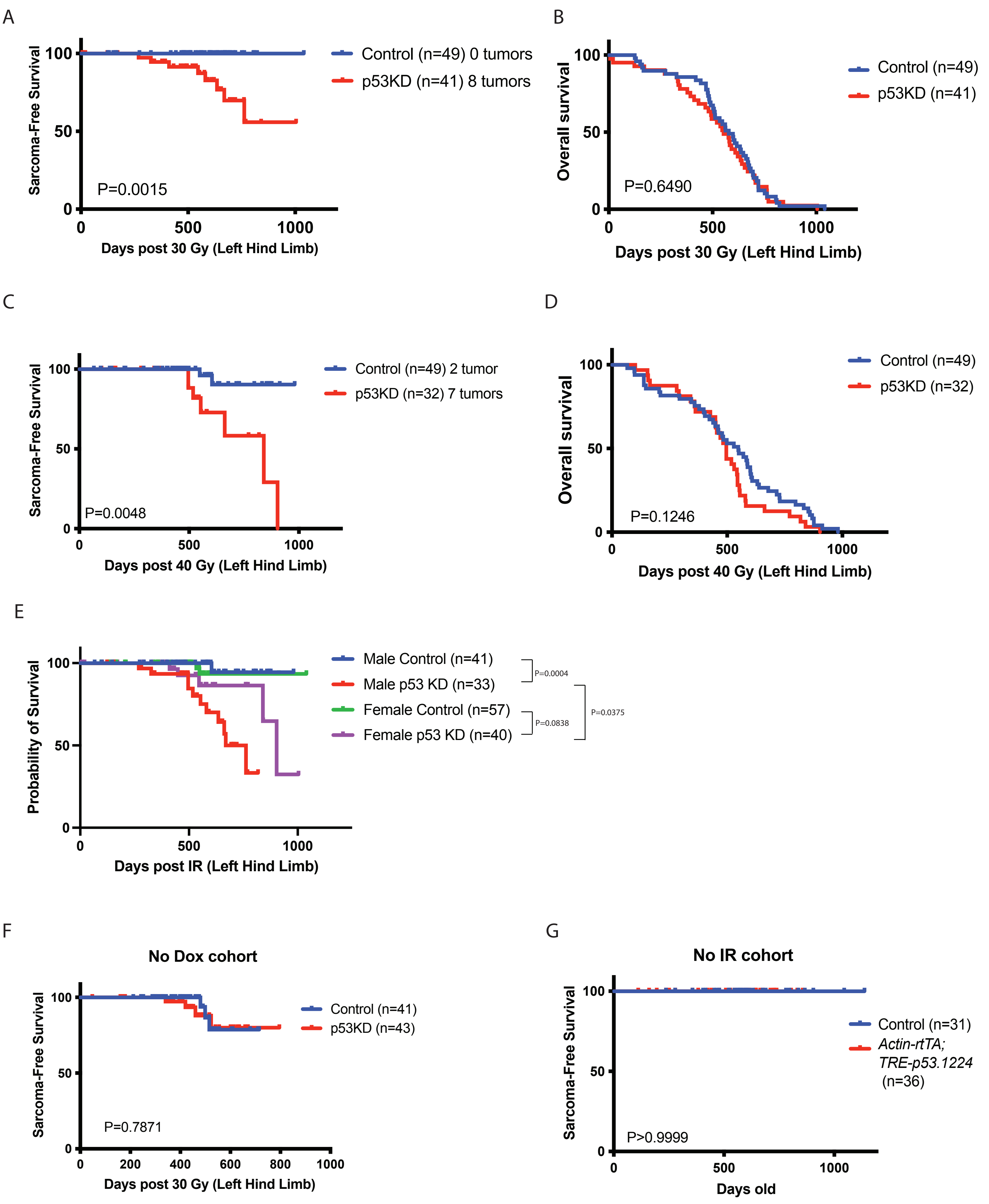

A
Sarcoma-Free Survival
100
50
0
P=0.0015
0
500
1000
Days post 30 Gy (Left Hind Limb)
Control (n=49) 0 tumors
p53KD (n=41) 8 tumors
B
Overall survival
100
50
0
P=0.6490
0
500
1000
Days post 30 Gy (Left Hind Limb)
Control (n=49)
p53KD (n=41)
C
Sarcoma-Free Survival
100
50
0
P=0.0048
0
500
1000
Days post 40 Gy (Left Hind Limb)
Control (n=49) 2 tumor
p53KD (n=32) 7 tumors
D
Overall survival
100
50
0
P=0.1246
0
500
1000
Days post 40 Gy (Left Hind Limb)
Control (n=49)
p53KD (n=32)
E
Probability of Survival
100
50
0
0
500
1000
Days post IR (Left Hind Limb)
Male Control (n=41)
Male p53 KD (n=33)
Female Control (n=57)
Female p53 KD (n=40)
P=0.0004
P=0.0838
P=0.0375
F
No Dox cohort
Sarcoma-Free Survival
100
50
0
P=0.7871
0
200
400
600
800
1000
Days post 30 Gy (Left Hind Limb)
Control (n=41)
p53KD (n=43)
G
No IR cohort
Sarcoma-Free Survival
100
50
0
P>0.9999
0
500
1000
Days old
Control (n=31)
Actin-rtTA; TRE-p53.1224 (n=36)

Figure S2. Temporary reduction in p53 expression during irradiation increases sarcomagenesis. (A) Kaplan-Meier curves show radiation-induced sarcoma-free survival of control and p53KD mice irradiated with 30 Gy to the hind limb. (B) Kaplan-Meier curves show overall survival of control and p53KD mice irradiated with 30 Gy to the hind limb. (C) Kaplan-Meier curves show radiation-induced sarcoma-free survival of control and p53KD mice irradiated with 40 Gy to the hind limb. (D) Kaplan-Meier curves show overall survival of control and p53KD mice irradiated with 40 Gy to the hind limb. (E) Kaplan-Meier curves show radiation-induced sarcoma-free survival of control and p53KD male and female mice irradiated with 30 or 40 Gy to the hind limb. P–values are from a log-rank test. (F) Kaplan-Meier curves show radiation-induced sarcoma-free survival of control and *Actin-rtTA; TRE-p53.1224* mice that did not receive dox and were irradiated with 30 Gy to the hind limb. (F) Kaplan-Meier curves show hind limb sarcoma-free survival of unirradiated control and *Actin-rtTA; TRE-p53.1224* mice. P –values are from log-rank tests.
