# Supplementary material for: Temporary Knockdown of p53 During Focal Limb Irradiation Increases the Development of Sarcomas
Source: Cancer Res Commun. 2023 Dec 5;3(12):2455–67. doi: 10.1158/2767-9764.CRC-23-0104 (PMC10697056; doi:10.1158/2767-9764.CRC-23-0104)
Supplement: Figure S3 — Supplementary figure S3 shows histological examination of mouse radiation-induced injuries [file crc-23-0104-s03.pdf]

Figure S3

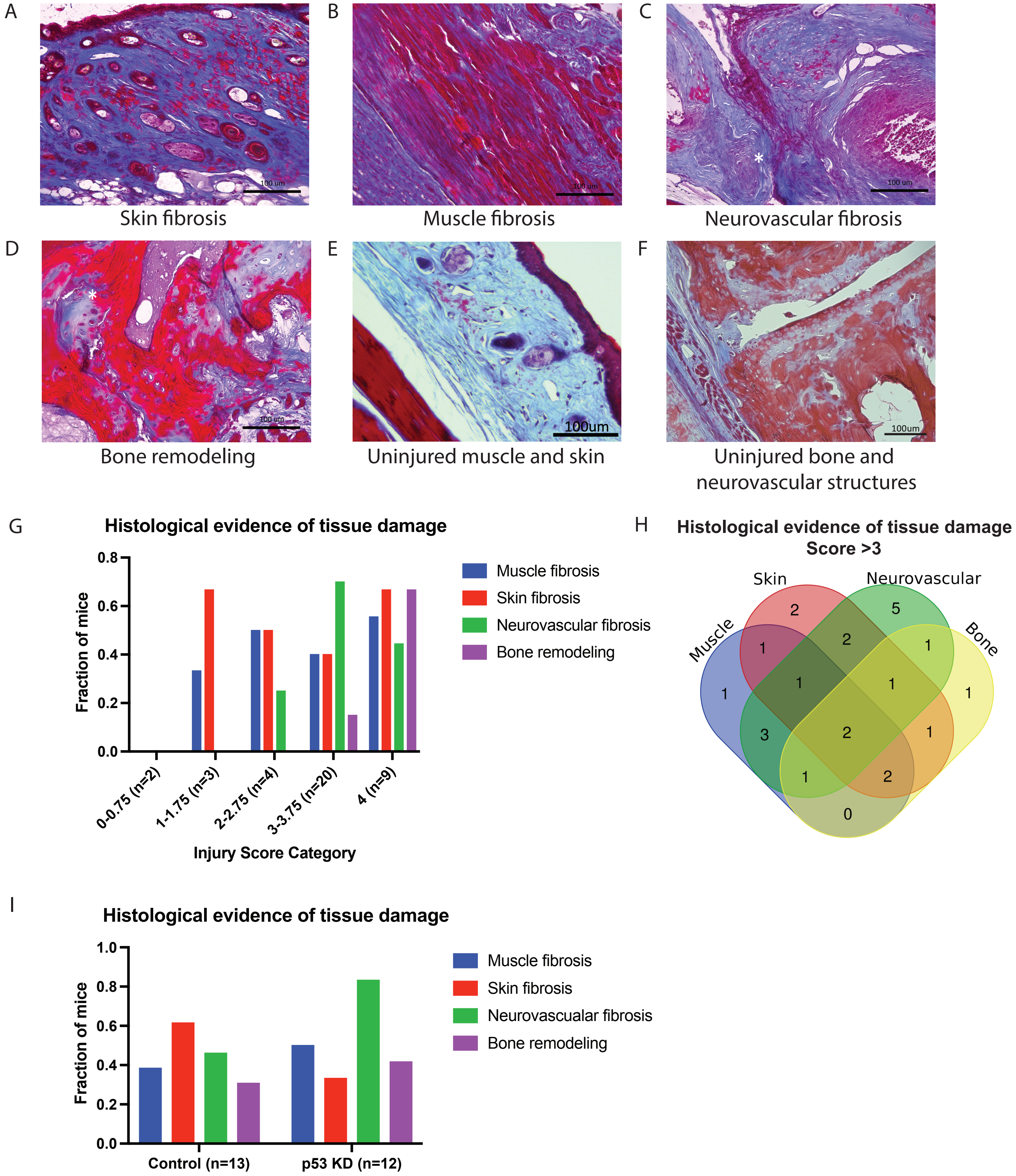
A
100 um
Skin fibrosis
B
100 um
Muscle fibrosis
C
100 um
Neurovascular fibrosis
D
100 um
Bone remodeling
E
100um
Uninjured muscle and skin
F
100um
Uninjured bone and neurovascular structures
G
Histological evidence of tissue damage
Fraction of mice
0.0
0.2
0.4
0.6
0.8
0-0.75 (n=2)
1-1.75 (n=3)
2-2.75 (n=4)
3-3.75 (n=20)
4 (n=9)
Injury Score Category
Muscle fibrosis
Skin fibrosis
Neurovascular fibrosis
Bone remodeling
H
Histological evidence of tissue damage
Score >3
Skin
Neurovascular
Muscle
Bone
2
5
1
2
1
1
1
1
1
3
2
1
1
2
0
I
Histological evidence of tissue damage
Fraction of mice
0.0
0.2
0.4
0.6
0.8
1.0
Control (n=13)
p53 KD (n=12)
Muscle fibrosis
Skin fibrosis
Neurovascualar fibrosis
Bone remodeling

Figure S3. Histological examination of mouse radiation-induced injuries. (A-D) Representative histological image of trichrome-stained radiation-induced skin fibrosis (A), muscle fibrosis (B), neurovascular fibrosis (C, *fibrotic nerve), and bone remodeling (D, * bone-cartilage junction). (E-F) Representative images of uninjured muscle and skin (E) and bone and neurovascular structures (F). (G) Bar graph representing the fraction of mice from each injury score category with histological evidence of skin, muscle, neurovascular, and bone injuries. Note, some mice have injuries in more than one category. (H) Venn diagram representing the number of control and p53KD mice with injuries scores greater than 3 with injuries to multiple tissue types by histological analysis. (I) Bar graph representing the fraction of control and p53KD mice with injury scores greater that 3 with histological evidence of skin, muscle, neurovascular, and bone injuries. Note, some mice have injuries in more than one category.
