# Supplementary material for: Temporary Knockdown of p53 During Focal Limb Irradiation Increases the Development of Sarcomas
Source: Cancer Res Commun. 2023 Dec 5;3(12):2455–67. doi: 10.1158/2767-9764.CRC-23-0104 (PMC10697056; doi:10.1158/2767-9764.CRC-23-0104)
Supplement: Figure S4 — Supplementary figure S4 shows high dose irradiation induces chronic injuries in mouse hind limbs [file crc-23-0104-s04.pdf]

Figure S4

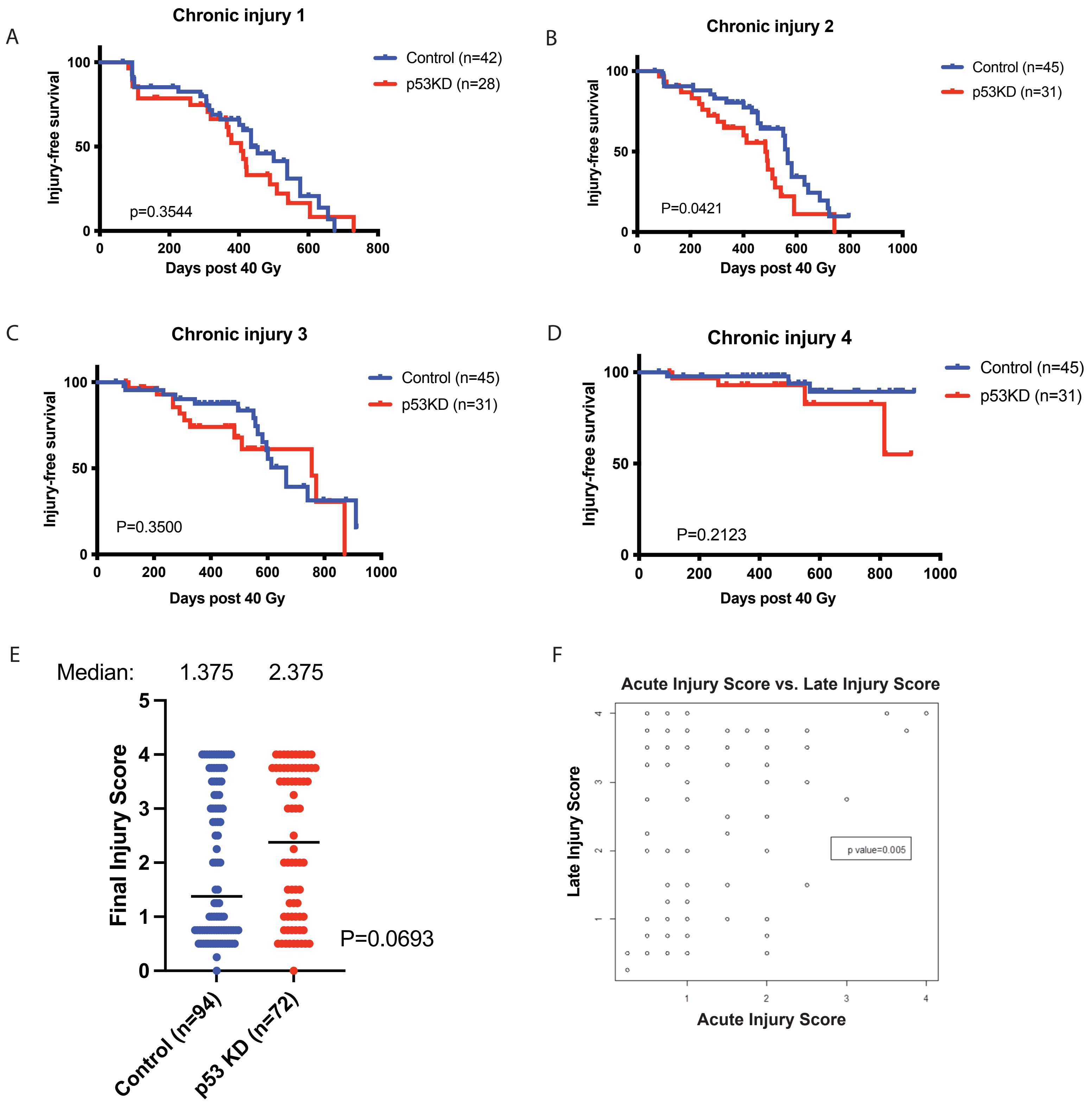

A
Chronic injury 1
Injury-free survival
Days post 40 Gy
Control (n=42)
p53KD (n=28)
p=0.3544
B
Chronic injury 2
Injury-free survival
Days post 40 Gy
Control (n=45)
p53KD (n=31)
P=0.0421
C
Chronic injury 3
Injury-free survival
Days post 40 Gy
Control (n=45)
p53KD (n=31)
P=0.3500
D
Chronic injury 4
Injury-free survival
Days post 40 Gy
Control (n=45)
p53KD (n=31)
P=0.2123
E
Median: 1.375 2.375
Final Injury Score
Control (n=94)
p53 KD (n=72)
P=0.0693
F
Acute Injury Score vs. Late Injury Score
Late Injury Score
Acute Injury Score
p value=0.005

Figure S4. High dose irradiation induces chronic injuries in mouse hind limbs. (A-D) Kaplan-Meier curves show chronic injury-free survival from scores 1+ (A), 2+ (B), 3+ (C), or 4 (D) of control and p53KD mice irradiated with 40 Gy to the hind limb. P–value is from a log-rank test. (E) The final injury scores of the control and p53KD mice that received 30 or 40 Gy are plotted. P-value is from a T-Test. (F) Correlation coefficient test compares acute injury scores and late (chronic) injury scores of the control and p53KD mice that received either 30 or 40 Gy to the hind limb. P-value was generated using a chi square test for the association between late score and acute score.
