# Supplementary material for: Temporary Knockdown of p53 During Focal Limb Irradiation Increases the Development of Sarcomas
Source: Cancer Res Commun. 2023 Dec 5;3(12):2455–67. doi: 10.1158/2767-9764.CRC-23-0104 (PMC10697056; doi:10.1158/2767-9764.CRC-23-0104)
Supplement: Figure S5 — Supplementary figure S5 shows mice without dox treatment sustained radiation-induced injuries in the hind limb [file crc-23-0104-s05.pdf]

Figure S5

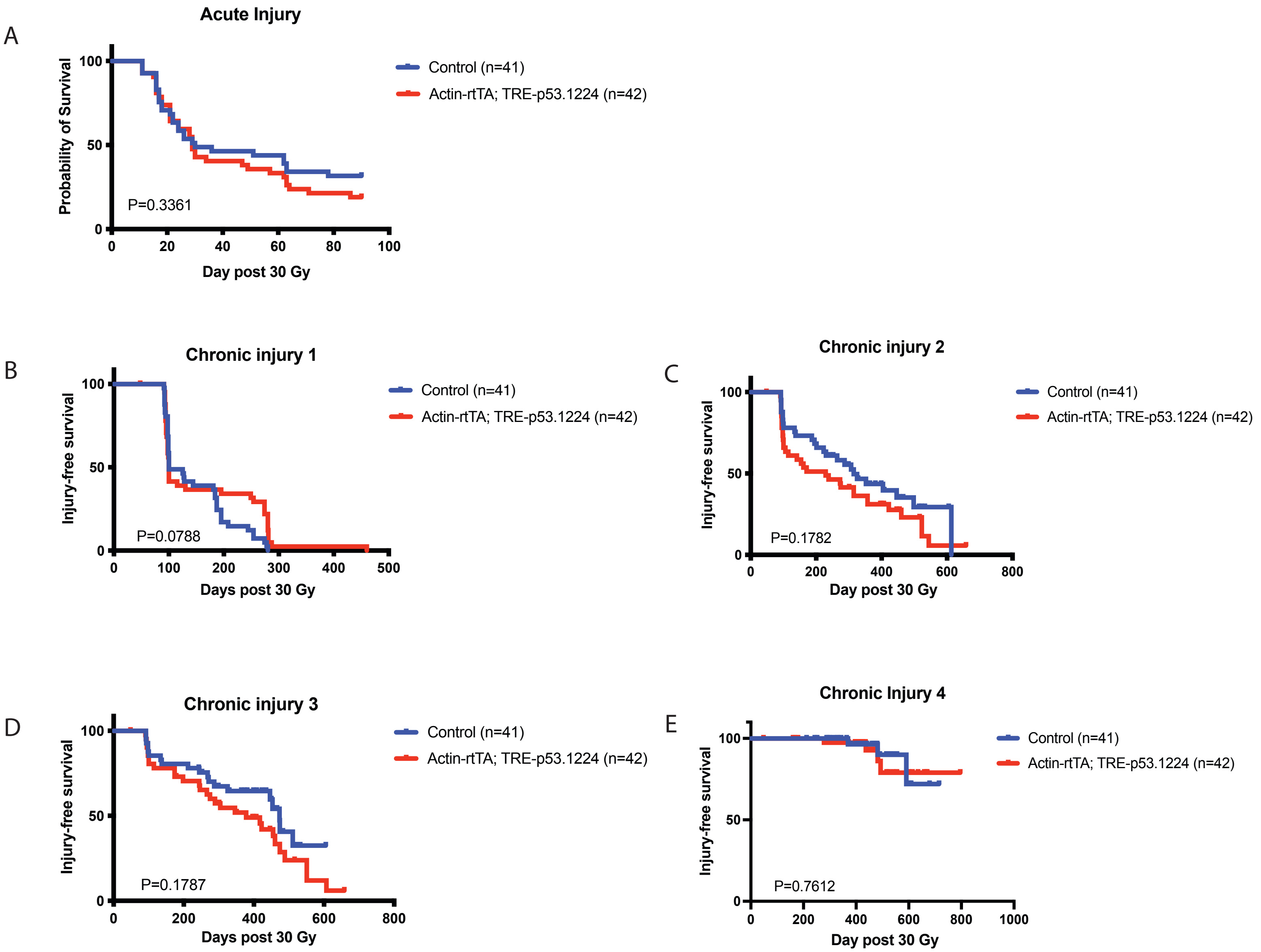
A
Acute Injury
Probability of Survival
100
50
0
0
20
40
60
80
100
Day post 30 Gy
P=0.3361
Control (n=41)
Actin-rtTA; TRE-p53.1224 (n=42)
B
Chronic injury 1
Injury-free survival
100
50
0
0
100
200
300
400
500
Days post 30 Gy
P=0.0788
Control (n=41)
Actin-rtTA; TRE-p53.1224 (n=42)
C
Chronic injury 2
Injury-free survival
100
50
0
0
200
400
600
800
Days post 30 Gy
P=0.1782
Control (n=41)
Actin-rtTA; TRE-p53.1224 (n=42)
D
Chronic injury 3
Injury-free survival
100
50
0
0
200
400
600
800
Days post 30 Gy
P=0.1787
Control (n=41)
Actin-rtTA; TRE-p53.1224 (n=42)
E
Chronic Injury 4
Injury-free survival
100
50
0
0
200
400
600
800
1000
Day post 30 Gy
P=0.7612
Control (n=41)
Actin-rtTA; TRE-p53.1224 (n=42)

Figure S5. **Mice without dox treatment sustained radiation-induced injuries in the hind limb.** (A) Kaplan-Meier curves show acute injury-free survival (score 1+) of control and *Actin-rtTA; TRE-p53.1224* mice irradiated with 30 Gy to the hind limb without prior dox treatment. P-value is from a log-rank test. (B-E) Kaplan-Meier curves show chronic injury-free survival from scores 1+ (B), 2+ (C), 3+ (D), or 4 (E) of control and *Actin-rtTA; TRE-p53.1224* mice irradiated with 30 Gy to the hind limb without prior dox treatment. P-value is from a log-rank test.
