# Supplementary material for: Temporary Knockdown of p53 During Focal Limb Irradiation Increases the Development of Sarcomas
Source: Cancer Res Commun. 2023 Dec 5;3(12):2455–67. doi: 10.1158/2767-9764.CRC-23-0104 (PMC10697056; doi:10.1158/2767-9764.CRC-23-0104)
Supplement: Figure S6 — Supplementary figure S6 shows that radiation-induced chronic injuries are associated with sarcomagenesis [file crc-23-0104-s06.pdf]

Figure S6

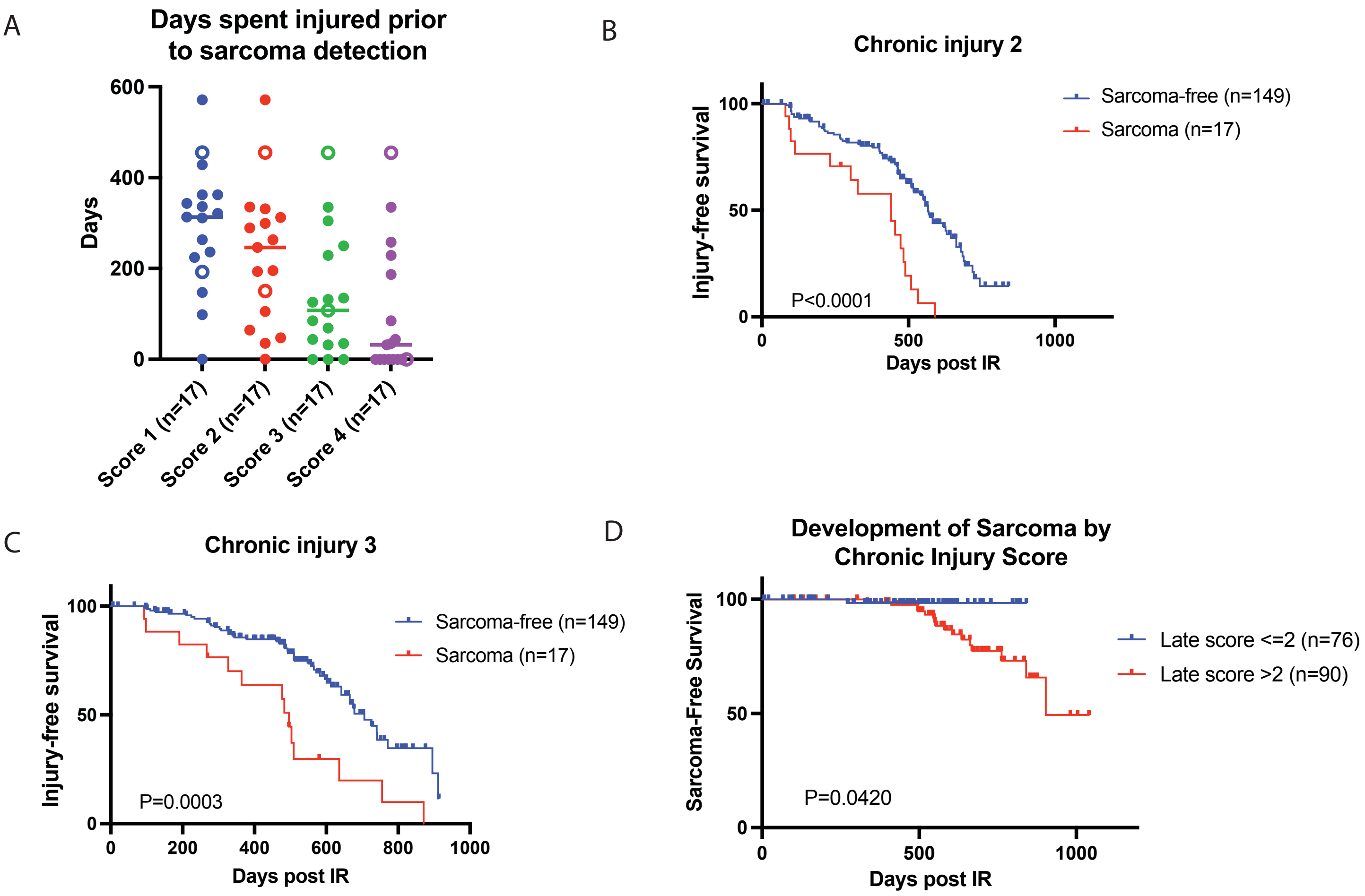
A
Days spent injured prior to sarcoma detection
Days
600
400
200
0
Score 1 (n=17)
Score 2 (n=17)
Score 3 (n=17)
Score 4 (n=17)
B
Chronic injury 2
Injury-free survival
100
50
0
0
500
1000
Days post IR
Sarcoma-free (n=149)
Sarcoma (n=17)
P<0.0001
C
Chronic injury 3
Injury-free survival
100
50
0
0
200
400
600
800
1000
Days post IR
Sarcoma-free (n=149)
Sarcoma (n=17)
P=0.0003
D
Development of Sarcoma by Chronic Injury Score
Sarcoma-Free Survival
100
50
0
0
500
1000
Days post IR
Late score <=2 (n=76)
Late score >2 (n=90)
P=0.0420

Figure S6. **Radiation-induced chronic injuries are associated with sarcomagenesis.** (A) Graph representing the number of days each mouse that developed radiation-induced sarcoma spent with injury scores 1+, 2+, 3+, or 4 prior to tumor detection. The open circles represent mice from the control group (n=2) and closed circles represent p53KD mice (n=15). (B-C) Kaplan-Meier curves show chronic injury-free survival from scores 2+ (B) or 3+ (C) of mice (control and p53KD) irradiated with 30 or 40 Gy to the hind limb that are sarcoma-free or radiation-induced sarcoma-bearing. P–value is from a log-rank test. (D) Kaplan-Meier curves show radiation-induced sarcoma-free survival of the control and p53KD mice irradiated with 30 or 40 Gy to the hind limb. Mice with chronic injury scores equal to or less than 2 are compared to mice with chronic injury scores greater than 2. P–value is from a log-rank test.
