# Supplementary material for: Temporary Knockdown of p53 During Focal Limb Irradiation Increases the Development of Sarcomas
Source: Cancer Res Commun. 2023 Dec 5;3(12):2455–67. doi: 10.1158/2767-9764.CRC-23-0104 (PMC10697056; doi:10.1158/2767-9764.CRC-23-0104)
Supplement: Figure S7 — Supplementary figure S7 shows gene expression analysis of radiation-induced sarcomas compared to normal muscles [file crc-23-0104-s07.pdf]

Figure S7

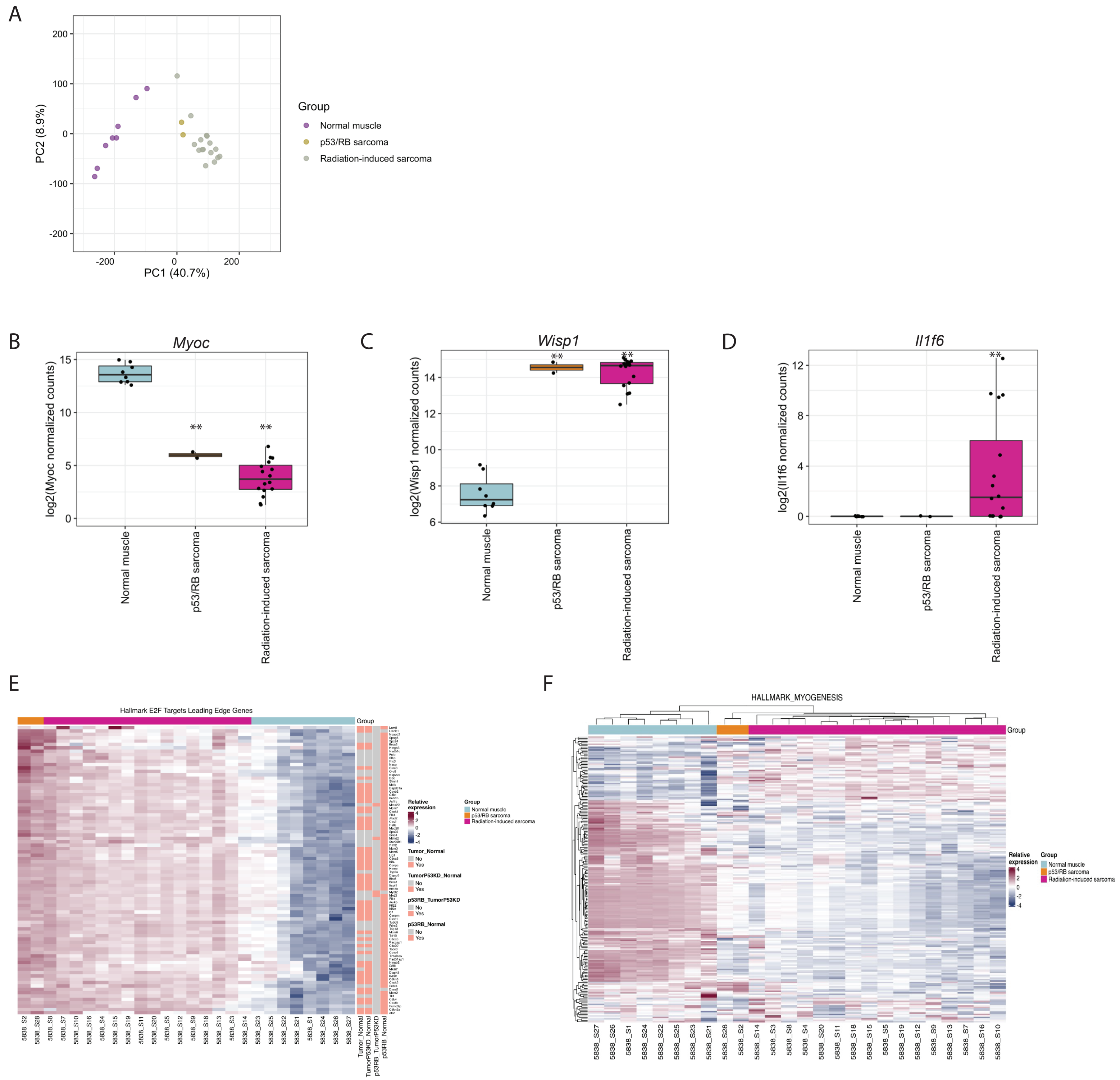

A
200
100
0
-100
-200
PC2 (8.9%)
-200
0
200
PC1 (40.7%)
Group
Normal muscle
p53/RB sarcoma
Radiation-induced sarcoma
B
Myoc
log2(Myoc normalized counts)
15
10
5
0
**
**
Normal muscle
p53/RB sarcoma
Radiation-induced sarcoma
C
Wisp1
log2(Wisp1 normalized counts)
14
12
10
8
6
**
**
Normal muscle
p53/RB sarcoma
Radiation-induced sarcoma
D
Il1f6
log2(Il1f6 normalized counts)
12
8
4
0
**
Normal muscle
p53/RB sarcoma
Radiation-induced sarcoma
E
Hallmark E2F Targets Leading Edge Genes
Group
Relative expression
4
2
0
-2
-4
Group
Normal muscle
p53/RB sarcoma
Radiation-induced sarcoma
Tumor_Normal
No
Yes
TumorP53KD_Normal
No
Yes
p53RB_TumorP53KD
No
Yes
p53RB_Normal
No
Yes
5838_S2
5838_S28
5838_S8
5838_S7
5838_S10
5838_S16
5838_S4
5838_S15
5838_S19
5838_S11
5838_S20
5838_S5
5838_S12
5838_S9
5838_S18
5838_S13
5838_S3
5838_S14
5838_S23
5838_S25
5838_S22
5838_S21
5838_S1
5838_S24
5838_S26
5838_S27
Tumor_Normal
TumorP53KD_Normal
p53RB_TumorP53KD
p53RB_Normal
F
HALLMARK_MYOGENESIS
Group
Relative expression
4
2
0
-2
-4
Group
Normal muscle
p53/RB sarcoma
Radiation-induced sarcoma
5838_S27
5838_S26
5838_S1
5838_S24
5838_S22
5838_S25
5838_S23
5838_S21
5838_S28
5838_S2
5838_S14
5838_S3
5838_S8
5838_S4
5838_S20
5838_S11
5838_S18
5838_S15
5838_S5
5838_S19
5838_S12
5838_S9
5838_S13
5838_S7
5838_S16
5838_S10

Figure S7. Gene expression analysis of radiation-induced sarcomas compared to normal muscles. (A) Principal Component Analysis of the mouse radiation-induced sarcomas compared to p53/RB sarcomas and normal muscles. (B) Boxplot of the differential expression of the *Myoc* gene in tumors compared to normal muscle. (C) Boxplot of the differential expression of the *Wisp1* gene in tumors compared to normal muscle. (D) Boxplot of the differential expression of the *Il1f6* gene in tumors compared to normal muscle. **P value >0.0001. (E) Heatmap of all genes in a leading-edge gene subset in the Hallmark E2F target pathway for any differential expression model. Expression is scaled relative to each row (gene), and samples and genes are hierarchically clustered. Genes in each row are annotated according to their membership in the leading edge of Hallmark E2F genes for each independent GSEA analysis (peach=in leading edge for a particular analysis, grey=not in leading edge). Samples are annotated by group (Normal, radiation-induced sarcoma, and p53/RB sarcoma samples). (F) Heatmap of all genes in the Hallmark Myogenesis target pathway. Expression is scaled relative to each row (gene) and genes are hierarchically clustered. Samples are annotated by group (Normal, radiation-induced sarcoma, and p53/RB sarcoma samples).
