# Supplementary material for: Temporary Knockdown of p53 During Focal Limb Irradiation Increases the Development of Sarcomas
Source: Cancer Res Commun. 2023 Dec 5;3(12):2455–67. doi: 10.1158/2767-9764.CRC-23-0104 (PMC10697056; doi:10.1158/2767-9764.CRC-23-0104)
Supplement: Figure S8 — Supplementary figure S8 shows immune infiltration analysis of radiation-induced sarcomas [file crc-23-0104-s08.pdf]

Figure S8

A

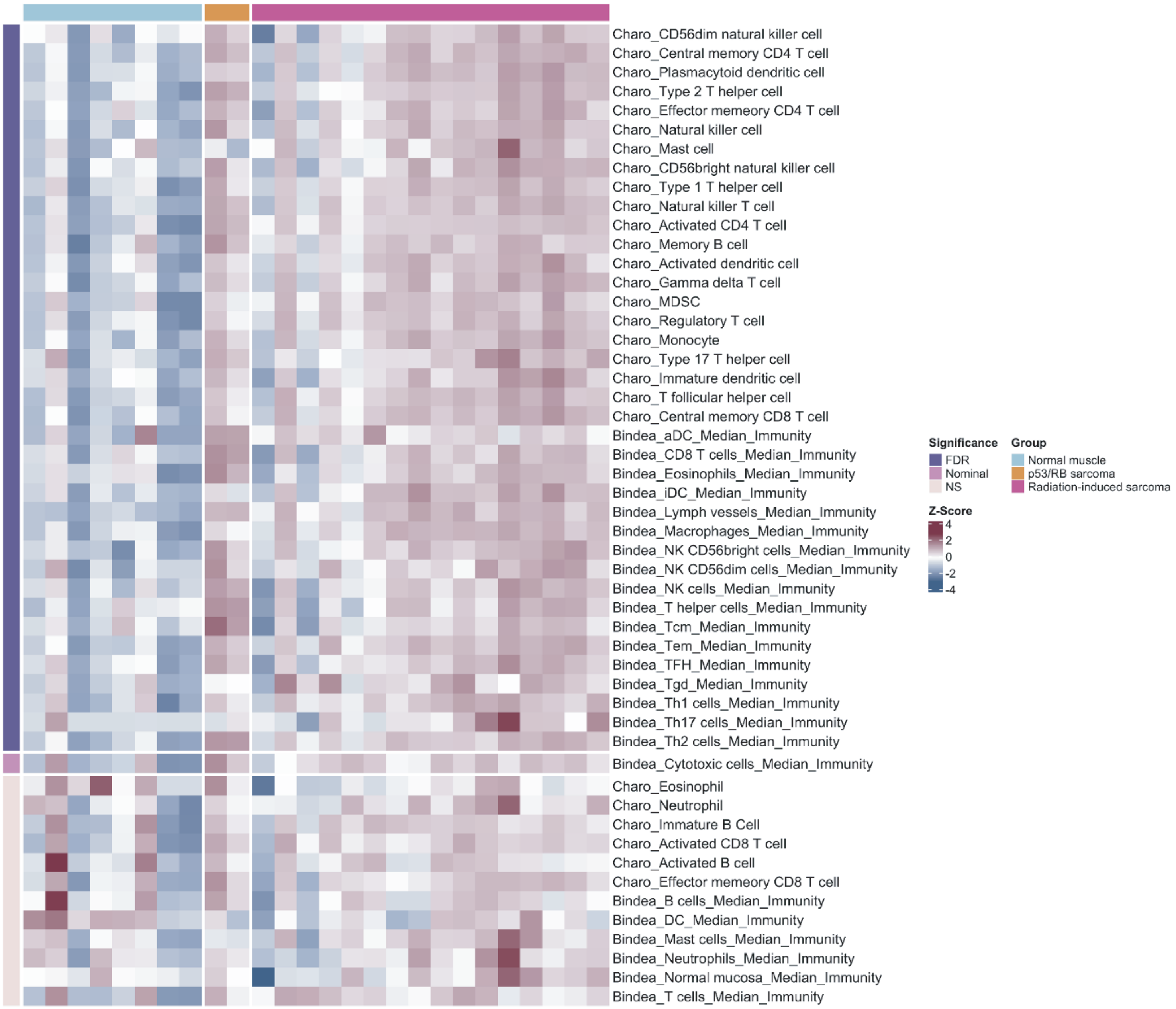
Charo_CD56dim natural killer cell
Charo_Central memory CD4 T cell
Charo_Plasmacytoid dendritic cell
Charo_Type 2 T helper cell
Charo_Effector memeory CD4 T cell
Charo_Natural killer cell
Charo_Mast cell
Charo_CD56bright natural killer cell
Charo_Type 1 T helper cell
Charo_Natural killer T cell
Charo_Activated CD4 T cell
Charo_Memory B cell
Charo_Activated dendritic cell
Charo_Gamma delta T cell
Charo_MDSC
Charo_Regulatory T cell
Charo_Monocyte
Charo_Type 17 T helper cell
Charo_Immature dendritic cell
Charo_T follicular helper cell
Charo_Central memory CD8 T cell
Bindea_aDC_Median_Immunity
Bindea_CD8 T cells_Median_Immunity
Bindea_Eosinophils_Median_Immunity
Bindea_iDC_Median_Immunity
Bindea_Lymph vessels_Median_Immunity
Bindea_Macrophages_Median_Immunity
Bindea_NK CD56bright cells_Median_Immunity
Bindea_NK CD56dim cells_Median_Immunity
Bindea_NK cells_Median_Immunity
Bindea_T helper cells_Median_Immunity
Bindea_Tcm_Median_Immunity
Bindea_Tem_Median_Immunity
Bindea_TFH_Median_Immunity
Bindea_Tgd_Median_Immunity
Bindea_Th1 cells_Median_Immunity
Bindea_Th17 cells_Median_Immunity
Bindea_Th2 cells_Median_Immunity
Bindea_Cytotoxic cells_Median_Immunity
Charo_Eosinophil
Charo_Neutrophil
Charo_Immature B Cell
Charo_Activated CD8 T cell
Charo_Activated B cell
Charo_Effector memeory CD8 T cell
Bindea_B cells_Median_Immunity
Bindea_DC_Median_Immunity
Bindea_Mast cells_Median_Immunity
Bindea_Neutrophils_Median_Immunity
Bindea_Normal mucosa_Median_Immunity
Bindea_T cells_Median_Immunity
Significance
FDR
Nominal
NS
Group
Normal muscle
p53/RB sarcoma
Radiation-induced sarcoma
Z-Score
4
2
0
-2
-4

**Figure S8. Immune infiltration analysis of radiation-induced sarcomas.** (A) Heatmap showing radiation-induced sarcomas compared to normal muscles utilizing modules from Charoentong et al. (21) and Bindea et al. (20). Kruskal-Wallis test of scores was conducted across the groups. Module scores shown are Z-score transformed. Abbreviations: false discovery rate (FDR); not significant (NS)
