# Supplementary material for: Temporary Knockdown of p53 During Focal Limb Irradiation Increases the Development of Sarcomas
Source: Cancer Res Commun. 2023 Dec 5;3(12):2455–67. doi: 10.1158/2767-9764.CRC-23-0104 (PMC10697056; doi:10.1158/2767-9764.CRC-23-0104)
Supplement: Figure S9 — Supplementary figure S9 shows gene expression analysis of mouse and human sarcomas that arose in irradiated tissue compared to sporadic sarcomas [file crc-23-0104-s09.pdf]

Figure S9

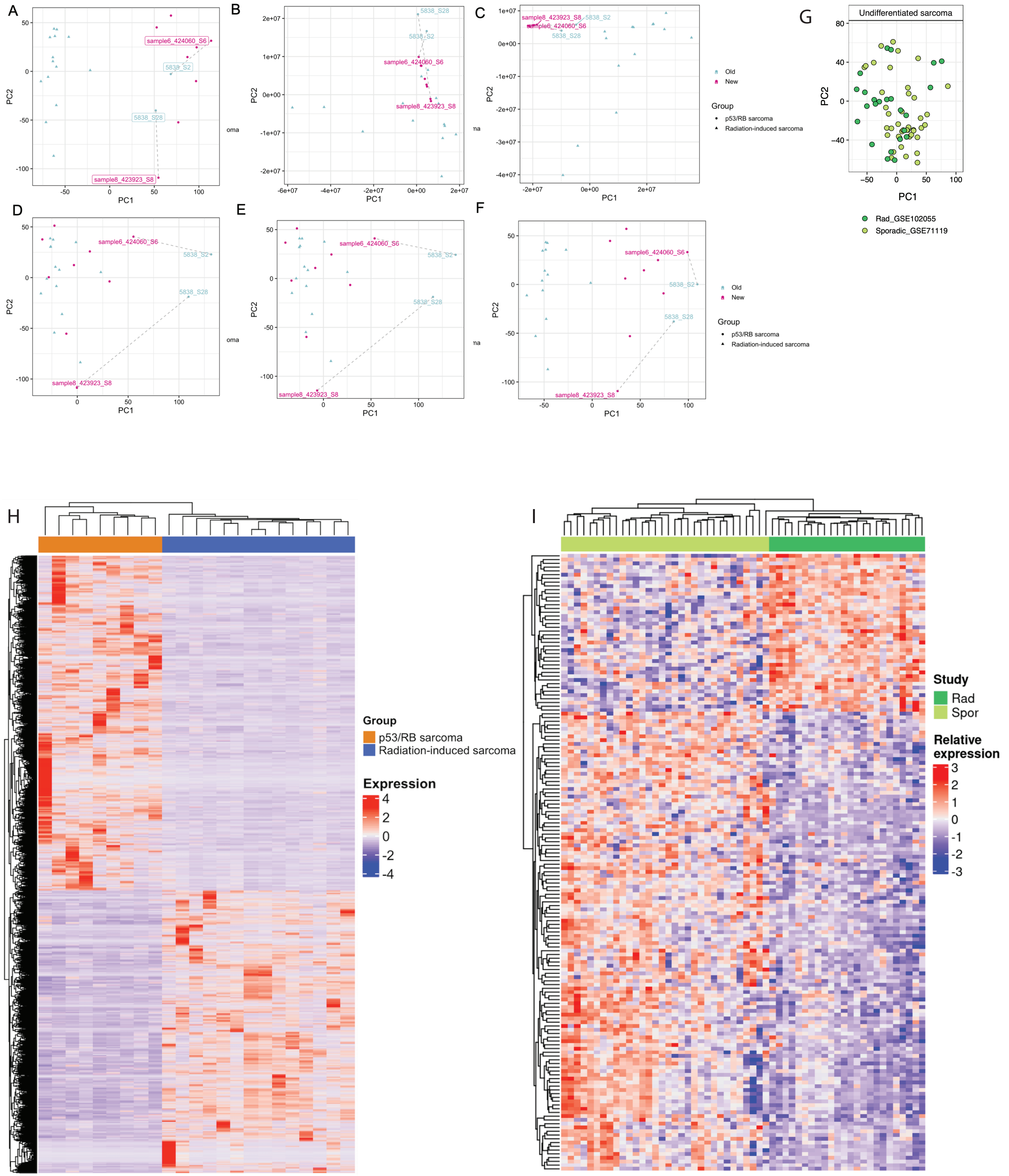
A
B
C
D
E
F
G
H
I
PC1
PC2
sample6_424060_S6
sample8_423923_S8
5838_S2
5838_S28
Old
New
Group
p53/RB sarcoma
Radiation-induced sarcoma
Undifferentiated sarcoma
Rad_GSE102055
Sporadic_GSE71119
Expression
Study
Rad
Spor
Relative expression

Figure S9. Gene expression analysis of mouse and human sarcomas that arose in irradiated tissue compared to sporadic sarcomas. (A-F) Scatter plots of the Principal Component (PC) 1 scores vs PC2 scores for the batch correction methods applied to the RNAseq data from mouse radiation-induced sarcomas and p53/RB sarcomas. Each dot represents one sample and the color represent the whether the sample was sequenced in the old batch (blue) or the new batch (pink). The dotted lines connect the matched samples between the old and new batches (A, Unadjusted; B, Combat adjusted; C, Combat-seq adjusted; D, Limma adjusted; E, median-adjusted; F, DWD adjusted). (G) Scatter plot of the PC1 scores vs PC2 scores for the RNAseq data from the human radiation-associated undifferentiated sarcomas (Rad) vs sporadic undifferentiated sarcomas (Sporadic). (H) Heatmap of the top differentially expressed genes in radiation-induced UPS (n=14) vs p53/RB sarcomas (n=9). Genes (rows) are colored by scaled, normalized expression values. Both rows and columns are clustered. (I) Heatmap of the top differentially expressed genes in the human radiation-associated undifferentiated sarcomas (n=24) vs sporadic undifferentiated sarcomas (n=42). Genes (rows) are colored by scaled, normalized expression values. Both rows and columns are clustered.
