# Supplementary material for: Temporary Knockdown of p53 During Focal Limb Irradiation Increases the Development of Sarcomas
Source: Cancer Res Commun. 2023 Dec 5;3(12):2455–67. doi: 10.1158/2767-9764.CRC-23-0104 (PMC10697056; doi:10.1158/2767-9764.CRC-23-0104)
Supplement: Figure S10 — Supplementary figure S10 show correspondence between gene expression and CNV in radiation-induced sarcomas [file crc-23-0104-s10.pdf]

Figure S10

A

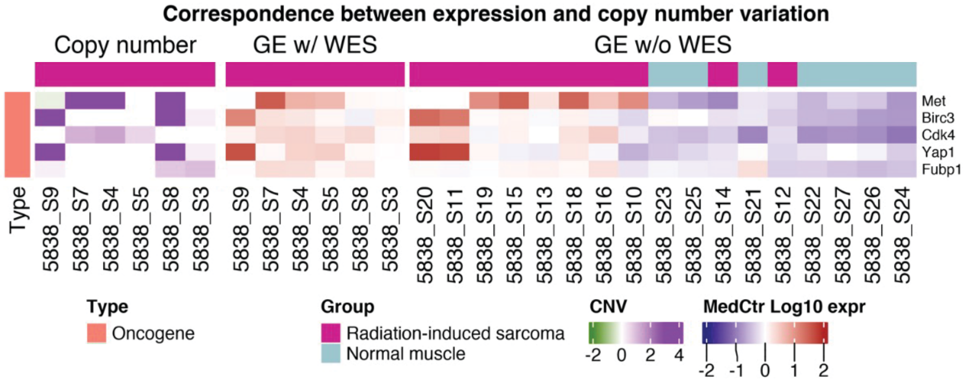
Correspondence between expression and copy number variation
Copy number
GE w/ WES
GE w/o WES
Met
Birc3
Cdk4
Yap1
Fubp1
Type
5838_S9
5838_S7
5838_S4
5838_S5
5838_S8
5838_S3
5838_S9
5838_S7
5838_S4
5838_S5
5838_S8
5838_S3
5838_S20
5838_S11
5838_S19
5838_S15
5838_S13
5838_S18
5838_S16
5838_S10
5838_S23
5838_S25
5838_S14
5838_S21
5838_S12
5838_S22
5838_S27
5838_S26
5838_S24
Type
Oncogene
Group
Radiation-induced sarcoma
Normal muscle
CNV
-2 0 2 4
MedCtr Log10 expr
-2 -1 0 1 2

Figure S10. Correspondence between gene expression and CNV in radiation-induced sarcomas. (A) Heatmap of COSMIC oncogenes with CNV by WES (9) (left, purple/green scale) and the gene expression (GE) values (right, red/blue scale) from the same tumors (middle, GE w/ WES). The GE from the remaining radiation-induced tumors and normal muscles that were not subjected to WES are plotted (right).
